# Human Papillomavirus (HPV) Infection-Associated Epithelial-to-Mesenchymal Transition: Implication in Tumorigenesis and Pathogenesis of HPV-Positive Cancers

**DOI:** 10.3390/cells15201916

**Published:** 2026-10-21

**Authors:** Alexander E. Kabakov, Olga N. Matchuk, Anna V. Khokhlova, Andrey D. Kaprin

**Affiliations:** 1 Department of Radiation Biochemistry, A. Tsyb Medical Radiological Research Center—Branch of the National Medical Research Radiological Center of the Ministry of Health of the Russian Federation, Koroleva St. 4, 249036 Obninsk, Russia; matchyk@mail.ru (O.N.M.);; 2 National Medical Research Radiological Center of the Ministry of Health of the Russian Federation, 249036 Obninsk, Russia; 3 Scientific and Educational Resource Center for Innovative Technologies of Immunophenotyping, Digital Spatial Profiling and Ultrastructural Analysis, Patrice Lumumba Peoples’ Friendship University of Russia (RUDN University), 117198 Moscow, Russia

**Keywords:** EMT, keratinocyte, malignant transformation, cancer stem cell-like cell, cell migration, invasiveness, metastasis, cervical cancer, head and neck cancer

## Abstract

Epithelial-to-mesenchymal transition (EMT) is a programmed and reversible process that allows epithelial cells to temporarily acquire a mesenchymal cell phenotype with increased migratory and invasive capacity. Analogous phenotypic modulation can also occur in carcinoma cells, which, after undergoing EMT, exhibit cancer stem cell-like properties and actively migrate, leading to tumor invasion and metastases. This structured narrative review focuses on the molecular crosstalk between human papillomavirus (HPV) and EMT, which occurs in HPV-infected human epithelium and in HPV-positive cancers of different location (cervical, head and neck, colorectal, lung, and others). The main questions addressed here are: (i) how HPV-driven EMT may contribute to the malignant transformation of HPV-infected epithelial cells, (ii) what mechanistic links may exist between the HPV positivity and EMT in human cancers, and (iii) how EMT in various HPV-positive human tumors may influence their pathogenesis, treatment modalities and outcomes. The relationship between HPV infection and EMT program is delineated as a complex multicomponent mechanism triggered by exogenous and endogenous stimuli and involving specific ligand-receptor interactions, membrane channels, transcription factors, signaling pathways, epigenetic regulators, etc. The review also discusses some of the potential applications of EMT-related biomarkers and molecular targets in the prevention, prediction and treatment of HPV-induced cancers.

## 1. Introduction

An estimated 21 million people are diagnosed with cancer annually, and the number is expected to increase by 77% by 2050 [1]. This is why enormous efforts are being directed towards the prevention or early diagnosis of malignant tumors and the development of new, more effective methods of antitumor therapy. Progress in these areas is impossible without a deep understanding of the mechanisms of tumorigenesis, as well as the factors that drive malignant growth and underlie tumor resistance to therapeutics.

This review is dedicated to the description and analysis of the relationships between human papillomavirus (HPV) infection and the phenomenon of the so-called epithelial-to-mesenchymal transition (EMT) in the context of HPV-associated oncogenesis and pathogenesis/treatment of HPV-positive tumors. A literature search was conducted using the PubMed/MEDLINE, Scopus, and Web of Science databases to identify publications concerning HPV-associated EMT in non-cancerous epithelial cells and cancer cells. The search covered the period up to August 2026 without start date; the final reference list included works published between 2003 and 2026. Search queries included combinations of the following terms: “EMT and HPV”, “EMT in HPV-infected epithelial cells/keratinocytes”, “EMT and transformation of HPV-infected epithelial cells/keratinocytes”, “EMT in HPV-induced lesions”, “HPV and cell migration”, “HPV, EMT, and oncogenesis”, “EMT in HPV-positive cancer”, “EMT in cervical cancer”, and “inhibitors of EMT”. Additional relevant publications were identified by reviewing the reference lists of the selected articles.

### 1.1. HPV: Structure, Life Cycle and Infectious/Carcinogenic Potential

HPV was discovered back in 1975, when it was first isolated from plantar warts. This is a non-enveloped double-stranded DNA virus, the genome of which consists of three regions: (i) the regulatory region (URR) containing the origin of replication, (ii) the early (*E*) gene region and (iii) the late (*L*) gene region. The *E* region encodes seven nonstructural proteins: E1, E2, E1ˆE4, E5, E6, E7, and E8ˆE2; the *L* region encodes two capsid proteins: L1 and L2 [2,3]. Due to the high variability of L1 protein, as of June 2024, the International Reference Center for HPV has identified 231 types of this virus [2].

**Figure 1:**
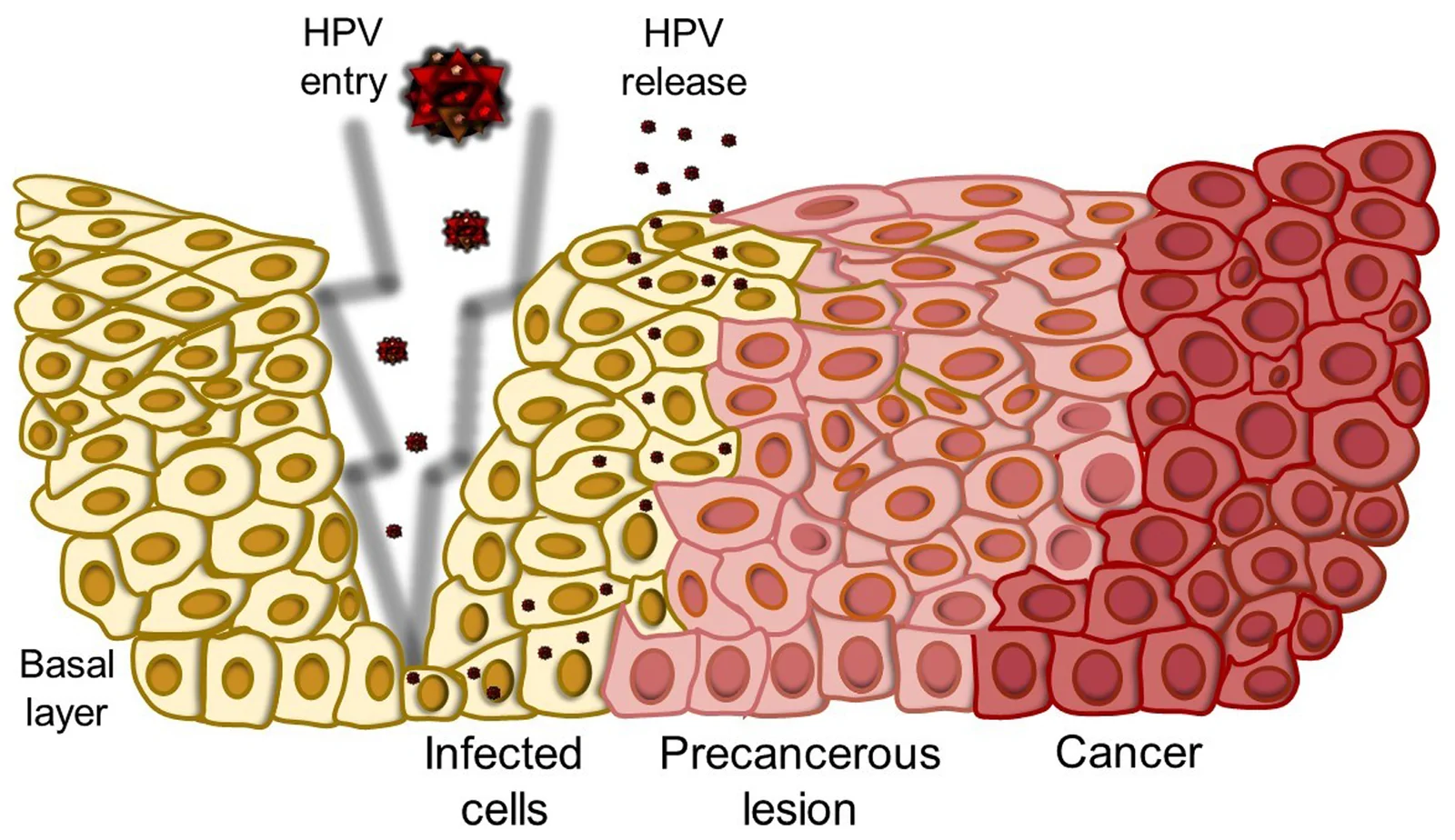
Simplified illustration showing HPV infection and its potential consequences: the virus attacks epithelial cells of the basal layer; infected cells then migrate upward, and after completing the viral life cycle, newly formed viral particles are released. If HPV persists, it creates a risk of developing precancerous lesions (pink zone) and cancer (red zone).

As a pathogen, HPV specifically attacks squamous epithelium of the skin or mucous membranes. Typically, HPV enters through micro-injuries on the epithelial surface, affecting the deeper and basal layers of epithelial cells (see Figure 1 and refs [2,3]). The virus initially binds to heparan sulfate proteoglycan on the cell surface and interacts with components of the extracellular matrix (ECM), which induces a conformational change in the viral capsid. This leads to viral entry into the target cell. Cell-penetrating HPV16 appears to be transported through early vesicles/endosomes, late endosomes/lysosomes, the Golgi apparatus, the endoplasmic reticulum, and finally enters the nucleus [2,3], although the exact mechanisms of this intracellular traffic remain to be determined.

After HPV enters the cell, viral replication begins. There are three phases in HPV life cycle: (i) establishment, (ii) maintenance, and (iii) production expansion. At the first stage (establishment), viral proteins E1 and E2 ensure amplification and regulation of the HPV genome. The protein E4, sometimes referred to as E1ˆE4, interacts with E2 to stabilize it, causes G2/M arrest, promotes *E6/E7* amplification, and maintains the intracellular mitogen-activated protein kinase (MAPK) in its active form. The presence of E4 is required for E5 protein expression, and both proteins contribute to viral DNA amplification. E5 is responsible for stimulating cell proliferation, re-entering the infected cells into the cell cycle, and triggering late viral functions [2,3]. At the phase of maintenance, HPV needs to maintain a constant pool of viral genomes and ensure a persistent infection. For this, E2 connects the viral genome to the host chromosome and promotes the partitioning of the viral genome into daughter cells during mitotic division. E8ˆE2, a protein product of *E2* spliced transcript that contains the hinge, DNA binding, and dimerization domains of E2, acts as a viral suppressor that limits HPV replication in undifferentiated cells [2,3]. During this phase, HPV DNA may integrate into the human genome. Such integration commonly disrupts both the *E1* and *E2* genes, which terminates the E2-dependent suppression of transcription of *E6* and *E7*, and can ultimately result in the malignant transformation of HPV-infected cells [2,3]. At the third phase (production expansion), a transition from stable maintenance of the HPV genome to its vegetative replication occurs in differentiated epithelial cells. The viral protein E6 is required for intraexosomal maintenance of the HPV genome during the viral life cycle, while the viral protein E7 specifically modulates functioning of multiple transcription factors in HPV-infected cells. The viral protein E5 functions to maintain host (HPV-infected) cells in a proliferation-competent state upon their differentiation. The capsid proteins L1 and L2 are expressed in highly or terminally differentiated epithelial cells and always after E4 expression. Each type of HPVs may have its own (unique) program of the late gene expression [2,3].

In most cases, HPV infection leads to harmless warts on the skin or mucous membranes. Nevertheless, the 2008 Nobel Prize in Physiology or Medicine was awarded to Harald zur Hausen for his discovery that HPV can provoke cervical cancer. Currently, all HPV types are classified as low- or high-risk depending on their pathogenicity and carcinogenicity. High-risk (oncogenic) HPV types are a cause of 5% of all human malignancies and are associated with cancers of the cervix and ano-genital area and also head and neck cancers [4]. In particular, HPV16 and HPV18 are considered to be responsible for approximately 70% of cervical cancer cases worldwide. In 2020, there were an estimated 604,000 new diagnoses of HPV-positive cervical cancer and 342,000 deaths worldwide [5]. Beta-human papillomaviruses (β-HPV) may be a cause of the development of non-melanoma skin cancers [6,7]. Evidence is accumulating about the implication of HPV infection in lung cancer [8]. It is believed that prolonged persistence of HPV in the infection loci can lead to the development of HPV-associated benign neoplasms, which, in turn, may subsequently transform into malignant tumors (see Figure 1 and Section 2.2).

HPV testing results hold significant prognostic value: many HPV-associated cancers are characterized by a more favorable clinical outcome compared to their HPV-negative anatomical counterparts [4]. The question of why HPV-positive tumors are associated with a more favorable prognosis is of considerable interest; however, there is currently no definitive answer. In head and neck carcinomas, HPV positivity has been found to be associated with the increased expression of parafibromin [9] and SMAD4 [10], which both are onco-suppressors and may therefore alleviate malignant growth and/or facilitate antitumor therapy. HPV integration into the genomes of cancer cells can impair their DNA repair system [11]. Compared with HPV-negative cancers, many HPV-positive oropharyngeal and cervical carcinomas exhibit increased radiosensitivity and are more susceptible to radiotherapy [11,12]. It can also be assumed that HPV-infected cancer cells are more effectively eliminated via immunogenic cell death following chemotherapy or radiation therapy. In vivo, the presence of HPV antigens among damage-associated molecular patterns (DAMP) released from dying cancer cells may enhance the subsequent immune response to a target (HPV-positive) tumor. Human-viral fusion peptides recently discovered in HPV-positive cervical cancer cells [13] may be recognized by cytotoxic T-lymphocytes as tumor-associated antigens.

However, the more favorable prognosis does not mean that all cases of HPV-related cancers are easily curable and non-lethal; unfortunately, the progression of HPV-positive tumors can be complicated by aggressive invasion, metastases, and chemoresistance, ultimately leading to death [4]. Consequently, developing methods to prevent HPV-associated carcinogenesis and improve the treatment efficacy for HPV-induced tumors is a pressing challenge.

While anti-HPV vaccination seems to be a rational and effective approach to prevent cervical cancer resulting from HPV infection [14], the problem of HPV-positive cancers is unresolved. In many countries, there are programs of cervical screening, including Pap smear tests, which help to detect both early malignancies and precancerous conditions that may develop into malignant ones [15]. Such screening allows HPV-infected individuals to be under surveillance aimed at early diagnosis and treatment; this strategy has reduced both the incidence and mortality of cervical cancers [15]. However, cases of HPV-related cancer still remain quite common and not easily curable [4]. The current situation with non-cervical HPV-related cancers is not more optimistic. Actually, the contribution of HPV infection to tumorigenesis and cancer pathogenesis requires further focused studies for developing appropriate strategies for prevention or therapy.

Section 2 and Section 3 of this review consider how the oncogenic properties of HPVs are associated with EMT in the virus-infected epithelial cells and how this association may influence the course of various cancers and treatment modalities.

### 1.2. EMT: Mechanisms, Stimuli, Drivers, and Role in Cancer Pathogenesis

In short, the phenomenon of EMT is a programmed (reversible) multi-step process, when epithelial cells lose their distinct phenotype and acquire the morphology and many of the features of mesenchymal cells (often fibroblast- or myofibroblast-like cells) with an increased capacity for migration and overproduction of the ECM [16,17].

**Figure 2:**
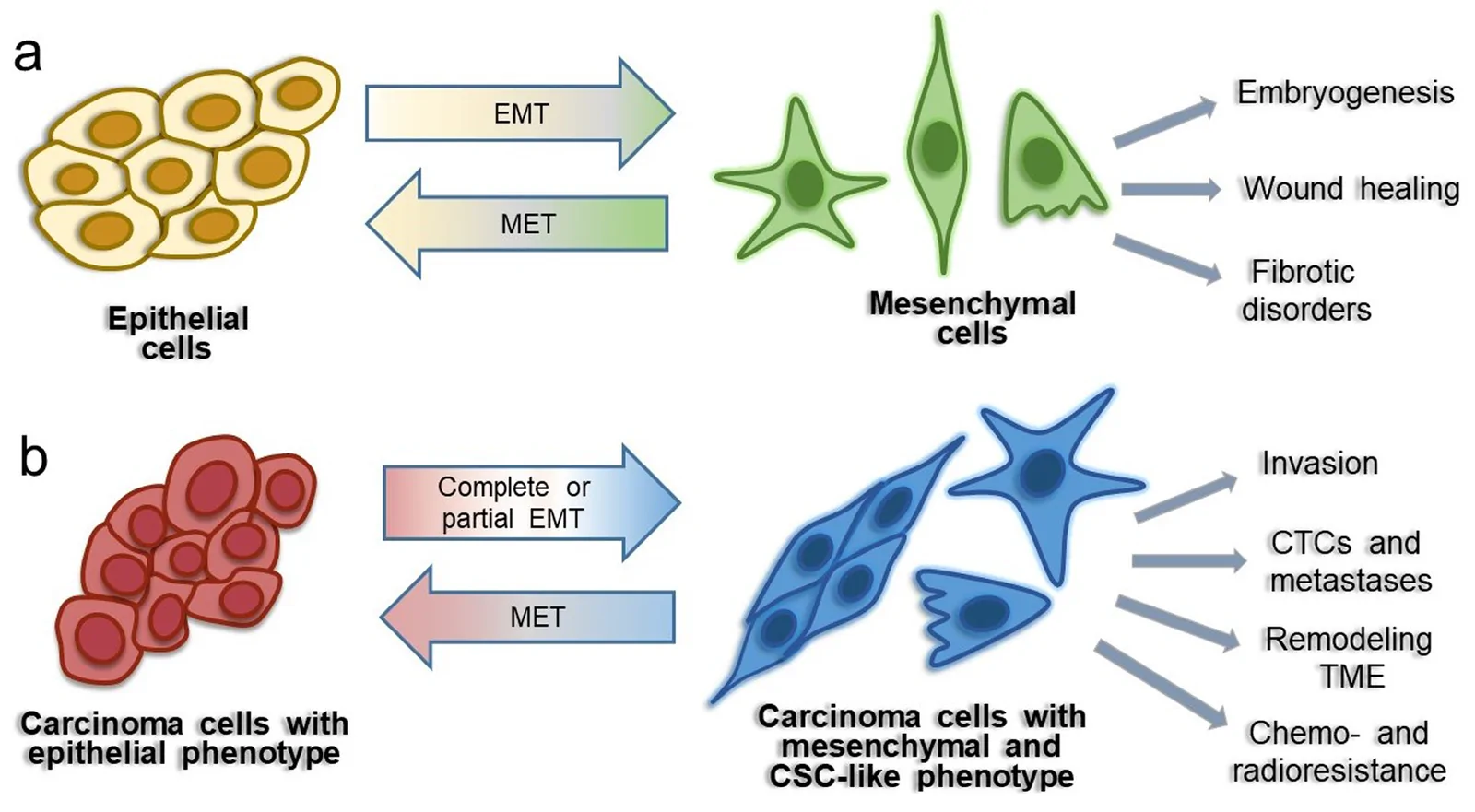
Simplified illustration showing EMT in normal epithelial cells (**a**) and carcinoma cells (**b**), including its physiological or pathological consequences. Abbreviations: EMT—epithelial-to-mesenchymal transition; MET—mesenchymal-to-epithelial transition; CTCs—circulating tumor cells; TME—tumor microenvironment; CSC—cancer stem cell.

EMT plays an important role in embryonic development, where this mechanism contributes to mesoderm formation and organogenesis. Moreover, EMT is involved in processes of tissue regeneration, when EMT is stimulated in sites of lesions to facilitate wound healing [16,17]. Due to the acquired contractile ability of myofibroblasts and the secretory activity of fibroblasts, cells that have undergone EMT are able to pull the wound edges together and fill the space with the newly produced ECM. However, deregulated EMT is known to contribute to excessive ECM production and the development of some pathologies such as fibrosis in the lungs, kidneys or liver, formation of keloids, cataracts, etc. (see Figure 2a and [16,17]). Furthermore, EMT can promote carcinogenesis (see Section 2) and, when stimulated in tumors, is a critical factor in cancer pathogenesis [16,18,19,20,21].

The prominent sign of epithelial cells undergoing EMT is the loss of their apical-basal polarity and cell–cell tight junctions that are characteristic for the epithelium. Instead, these cells acquire a new, (myo)fibroblast-like (spindle- or star-shaped) morphology, which is accompanied by a manifestation of new capabilities, such as a high motility and tendency to migrate, invasiveness, enhanced production of fibronectin and other ECM components (Figure 2). Importantly, the phenomenon of EMT can take place not only in the normal epithelium, but also in carcinomas (epithelium-derived malignancies), proceeding via similar molecular mechanisms [16,17,18,19,20]. In both normal epithelial cells and carcinoma cells, the EMT-associated alterations in cellular morphology/traits are due to the EMT-reprogrammed expression of a set of proteins, namely the repression of epithelial markers (e.g., E-cadherin, cytokeratins) and emergence of mesenchymal markers (N-cadherin, vimentin, alpha-smooth muscle actin (αSMA), others) [16,17,18,19,20]. Those alterations in the protein profiles are mainly caused by the EMT-driven changes in gene transcription that are regulated by transcriptional factors Snail, Slug, Twist, ZEB1/2 and others, while being triggered by a transforming growth factor beta (TGF-β)-pathway and/or other signaling pathways such as MAPK-, PI3K/Akt-, Wnt/β-catenin-, Notch-, hypoxia-inducing factor (HIF)-, Hedgehog-, and Ca^2+^-signaling [16,17,18,19,20]. There are epigenetic regulators of EMT in cancer, such as enzymes modifying DNA and histones, long non-coding RNAs, and microRNAs (miRNAs), that control the functions of key EMT-initiating transcription factors (Snail, Slug, Twist, and ZEB1/2) or interact with TGF-β- and Wnt/β-catenin-pathways [20,22]. Certain transmembrane proteins such as growth factor receptors and ion channels (in particular, Ca^2+^ channels) are implicated in initiation and regulation of EMT [23]. Also, programmed death receptor ligand 1 (PD-L1), frequently expressed in tumors, is one of the drivers of EMT, while the implemented EMT promotes the enhanced expression of PD-L1 that confers an immune-evasive capability on cancer cells [24,25].

Besides TGF-β, the other known external stimuli and promoters of EMT in the normal epithelium and tumors are: fibroblast growth factor (FGF), hepatocyte growth factor (HGF), inflammatory mediators (IL-6, TNF-α, IFN-γ), infectious agents (including HPV, see further in this paper), or their derivatives such as lipopolysaccharides (LPS), reactive oxygen species (ROS), and stressful conditions in the cellular microenvironment (hypoxia, acidosis, high ECM stiffness, etc.) (reviewed in [16,17,18,19,20,21]). In vivo, exosomes secreted by tumor cells are thought to be one of the stimuli of EMT, when exosomal cargo such as specific miRNA triggers EMT in recipient cancer cells [26]. Finally, some drugs [27,28] and radiation exposure [29,30] are also able to stimulate EMT in cancer cells, which, in some cases, may devalue the efficacy of antitumor chemotherapy and radiation therapy, if treatment-stimulated EMT promotes metastasis dissemination.

EMT plays an extremely important role in cancer pathogenesis [18,19,20,21]. In addition to complete EMT, partial or hybrid EMT can occur that is an intermediate phenotypic state, where cancer cells simultaneously express both epithelial and mesenchymal markers [19,31]. Instead of total E-cadherin loss, as occurs in complete EMT, cancer cells undergoing partial EMT can still express E-cadherin and/or P-cadherin along with expression of vimentin. Unlike complete phenotypic switching, partial (hybrid) EMT allows carcinoma cells to retain their attachment to each other while acquiring migratory properties which leads to the collective migration of multicellular clusters, more aggressive invasion, in-creased levels of circulating tumor cells (CTCs) and, consequently, increased metastatic spread (see Figure 2b and [19,31,32]). Moreover, the partial (hybrid) EMT in tumors is commonly associated with maintaining cancer stemness, weak responses to therapeutics and poor outcomes [19,31].

As a result of the EMT program implemented in tumors, some carcinoma cells are transformed into fibroblast-like, mobile malignant cells that actively migrate through the ECM/basal membranes and penetrate blood and lymphatic vessels, leading to tumor invasion into adjacent organs and an emergence of CTCs (Figure 2b). CTCs are the precursors of distant metastases, spreading through the blood and lymph circulation [33]. When metastases begin to grow in their metastatic niches, a reverse process occurs—the so-called mesenchymal-to-epithelial transition (MET), as a result of which carcinoma cells again acquire an epithelial phenotype [34]. Such phenotypic plasticity and EMT-MET alternations mainly determine the pathogenesis of metastatic cancers [34]. Moreover, EMT appears to play a pivotal role in the development and progression of carcinosarcomas—rare and particularly aggressive solid tumors that represent a mixture of malignant cells with epithelial and mesenchymal phenotypes (reviewed in [35,36]).

The significant capability to migration of the EMT-transformed cancer cells is due to a number of the phenotypical traits and activities inherent in mesenchymal malignant cells, including the critical alterations in intercellular contacts (replacing E-cadherin to N-cadherin or P-cadherin, relocalization of β-catenin, occludin, zona occludens-1 (ZO-1) and claudins), cytoskeleton (exchange of cytokeratins for vimentin, expression of αSMA, upregulated fascin and altered regulation/dynamics of actin filaments), cell-ECM interactions (the EMT-induced expression of certain isoforms of integrins and other cell surface receptors), biosynthesis and secretory activity (the EMT-enhanced production of angiogenic factors, ECM components and also enzymes that target ECM components), etc. [16,17,18,19,20,21]. For a malignant cell to be able to migrate quickly through the ECM, it must produce and secrete enzymes that torn apart ECM in front of the cell as it moves—this is exactly what cancer cells that have undergone EMT are capable of: they secrete proteases (in particular, metalloproteinases (MMPs) and plasminogen activator), hyaluronidase, heparanase, etc., creating a path for their migration through the basement membranes, ECM, and vascular walls, which leads to invasion and metastases [16,17,18,19,20,21].

The so-called vasculogenic mimicry is a specific form of intratumoral EMT, in which some fraction of cancer cells turns into cells with endothelial-like and pericyte-like (mesenchymal) phenotypes that build additional (false) capillaries and microvessels to improve tumor blood supply [37,38]. Such EMT-conferred pseudovascularization of tumors also promotes their growth and metastasis spread.

Importantly, having undergone EMT, tumor cells acquire many traits of cancer stem cells (CSCs), including their ability to renewal and their resistance to apoptosis, senescence, immunosurveillance, and also radiation exposure and cytotoxic drugs [39,40,41]. These EMT-generated CSC-like cells are able to survive chemotherapy and radiation therapy, causing tumor recurrence. The CSC-like tumor cells have characteristic phenotypic markers including specific cell surface antigens and upregulated expression/activities of certain enzymes and membrane transporters with a drug-pumping-out function. Oncogenes, epigenetic regulators, signaling pathways, transcriptional factors, and molecular chaperones are known to contribute to the development/maintenance of a CSC-like phenotype among tumor cells [39,40,41,42]. In particular, the Notch and Hedgehog signaling pathways promote self-renewal of CSC-like cells by driving the expression of NANOG, Slug, Twist, SOX2 and OCT4. The Wnt signaling, being hyperactivated in CSC-like cells, was shown to upregulate the expression of CD44, CD133, LGR5, aldehyde dehydrogenase (ALDH), and membrane transporter ABCG2 (all are phenotypic markers of CSCs) [39,40,41,42]. The important cancer-promoting feature of the EMT-generated CSC-like tumor cells is their angiogenic potential; indeed, these cells produce vascular endothelial growth factor (VEGF), thereby stimulating tumor vascularization and malignant growth [43].

In addition to the contribution of cancer cells with EMT-modulated phenotypes to the tumor vascularization [37,38,43], these cells actively remodel the tumor microenvironment (TME), making it immunosuppressive [44]. This is achieved through their ability to secrete certain cytokines and chemokines, certain ECM components, ECM-affecting enzymes, and other factors that (i) recruit and stimulate fibroblasts, thus creating an abundant pool of cancer-associated fibroblasts (CAFs), and (ii) reprogram and misdirect macrophages and other immune cells so that the EMT-remodeled TME enables the tumor to evade the host immune response [44].

To summarize all of the above, intratumoral EMT creates a very specific fraction of “extremely malignant cells” that have the ability to evade immune attacks, self-renew, invade and metastasize, remodel the TME and build an additional vascular network, and resist therapeutics (Figure 2b). Therefore, the consequences of intratumoral EMT make this phenomenon one of the main problems in cancer treatment. The next sections present data on the interplay between HPV and EMT and show how these data may be used for lowering the risk of HPV-induced cancers and also for prognosis and therapy of patients with HPV-positive tumors.

## 2. Implication of HPV-Associated EMT in Transformation of Human Epithelial Cells and Development of Precancerous Lesions

### 2.1. EMT in Epithelial Cells Transformed by HPV Infection or Expression of Certain HPV Genes

#### 2.1.1. Characteristic Alterations in the Morphology and at the Molecular Level Caused by HPV Infection or Expression of HPV E6/E7 Genes in Human Epithelial Cells

The ability of HPV to cause immortalization of the cultures of human skin or gingival keratinocytes was established many years ago. The HPV-conferred immortalization of epithelial cells is thought to be one of the essential steps in the process of their neoplastic transformation; however, it seems very important that the transformation of HPV16/18-infected epithelial cell cultures was usually accompanied by EMT [45,46,47,48,49,50]. Likewise, HPV31 infection in a culture of human gingival keratinocytes immortalized by human telomerase reverse transcriptase (hTERT) was shown to induce EMT in those cells [51].

Human keratinocyte cultures, being immortalized by stable transfection with HPV *E6/E7* genes, also exhibited EMT with its hallmarks [52,53,54,55,56]. In those in vitro studies, the HPV-associated EMT was clearly manifested in (i) the characteristic changes in the morphology of the keratinocyte monolayer (acquisition of a spindle-like cell shape and loss of tight cell–cell junctions), (ii) the replacement of epithelial molecular markers (cytokeratins and E-cadherin) by mesenchymal ones (vimentin and N-cadherin, respectively), and (iii) the substantial increase in the cell migratory and invasive potential.

An intriguing question arises: what is the molecular mechanism that triggers the EMT program in HPV-infected keratinocytes? In other words, how HPV infection induces the expression and activation of the key EMT drivers (Twist, Snail, Slug, ZEB1/2) in the infected keratinocytes? This issue is not yet fully clarified, but several multi-step and multi-factor mechanisms have been discussed and suggested herein. For instance, the molecular axis HPV E6/p53/miR-34a/Snail may be responsible for the induction of EMT in HPV-infected cells [57]. Mechanistically, it has been suggested that E6 binds p53, causing its ubiquitin-dependent degradation, and then the loss of p53 leads to the downregulation of miR-34a, while miR-34a is known to be an endogenous suppressor of Snail; as a result, E6 provides Snail activation in HPV-infected cells followed by Snail-induced EMT and EMT-driven cell migration [57].

Xu et al. [47,48] revealed that EMT in HPV-immortalized human keratinocytes is mediated by the overexpression of the transcriptional factor Six1 which leads to an increase in the TGF-β receptor type II expression and stimulation of the MAPK pathway (both events promote EMT). Furthermore, the researchers found that Six1 overexpression in HPV-immortalized human keratinocytes results in the generation of the CSC-like cell population (one of the consequences of EMT) and completes their malignant conversion [47]. Later, a contribution of Six1 to the progression of HPV16-transformed human keratinocytes toward EMT and tumorigenicity was confirmed by another publication [49].

HPV E2 protein appears to be able to prevent both the EMT-inducing effects of HPV16 and the carcinogenicity attributed to this virus. Fontan et al. [58] reported that HPV16 E2 expression in human skin keratinocytes (N/Tert-1 line) downregulates TWIST1 mRNA and protein levels in them and inhibits their migratory activity in wound-healing test. This finding may explain why the loss of the *E2* gene during HPV integration into the human genome promotes HPV-associated carcinogenesis and also suggests a carcinogenic effect of EMT that occurs at loci of HPV infection.

In a recent study, Tan et al. [50] reported that HPV16 and HPV18 infections significantly enhance the expression of transient receptor potential canonical channel 3 (TRPC3, a Ca^2+^ channel) in immortalized cell lines and primary cultures of human cervical epithelial cells, which was accompanied by EMT with the expression of ZEB1, Snail, Slug and Twist1. Mechanistically, it was due to TRPC3-mediated Ca^2+^ influx that modulated Ras-MAPK and MEK-ERK signaling pathways, thereby maintaining the viability and EMT-conferred phenotype in the HPV-infected cells. The depletion or inhibition of TRPC3 effectively suppressed both the Ca^2+^ influx and HPV-associated EMT [50].

The overexpression of NADPH oxidase homolog 1 (Nox1), a ROS-generating enzyme, was shown to induce EMT in HPV16 E6/E7-immortalized human gingival keratinocytes, which conferred them a spindle-like cell shape, EMT-related alterations in the cytoskeleton (appearance of vimentin and αSMA, and others), and an ability to anchorage-independent growth along with enhanced migration and invasiveness [54]; those authors suggested that Nox1 accelerates neoplastic transformation in HPV-infected human epithelial cells via EMT induction in them.

In a three-dimensional invasive organotypic raft culture model, human foreskin keratinocytes expressing *E6/E7* genes of HPV16 acquired invasiveness and exhibited SNAIL2 (Slug)-mediated EMT with downregulation of E-cadherin and upregulation of vimentin and Slug [55]. This HPV16 E6/E7-driven mechanism of EMT/invasion in cultured human keratinocytes was found to involve Src kinase signaling and be sensitive to the in vitro action of dasatinib, an inhibitor of tyrosine kinases including Src kinase [55].

Wang et al. [56] have demonstrated that the overexpression of hTERT (*HERT* + *HERC* genes) in E6/E7-immortalized human keratinocytes induced EMT in them, which was manifested in the expression of vimentin, N-cadherin, ZEB1 and MMP2 along with the decrease in cytokeratin and E-cadherin. It seems important that those E6/E7-immortalized human keratinocytes, having undergone the TERT/TERC overexpression-mediated EMT, became anchorage-independent and tumorigenic in immunodeficient mice [56]; this provides further evidence of a relationship between EMT and malignant transformation in HPV-infected epithelial cells.

In normal keratinocytes of human skin-derived cell line HaCaT, the overexpression of both HPV16 E6 and E7 oncoproteins was shown to enhance the expression of PiwiL1, a protein regulating stem cell renewal and RNA silencing, while excess PiwiL1 in HaCaT cells conferred the upregulation of molecular signatures of EMT (N-cadherin, vimentin, Snail2, Twist1/2, MMP9) and stemness (NANOG, OCT4, CD44, ABCG2) [59]. In support of these in vitro data, PiwiL1-overexpressing HaCaT cells that acquired traits of a mesenchymal and stem-like phenotype exhibited increased migratory, invasive, and tumorigenic abilities in nude mice [59].

#### 2.1.2. HPV-Driven Transformation of Human Epithelial Cells Facilitates EMT Induction in Them Under the Influence of Other (External) Factors

Importantly, (pro-)inflammatory mediators appear to be able to trigger EMT in the HPV-transformed human epithelium. In support of this, Hong et al. [60] observed an increase in the EMT-conferred phenotypes in HPV-immortalized human oral keratinocytes in response to long-term treatments with tumor necrosis factor α (TNFα), the major pro-inflammatory cytokine. In that case, TNFα-induced EMT was manifested by a decrease in the expression of genes involved in the formation of cell junctions and an increase in migratory capacity, as well as the activation of Notch signaling and the acquisition of CSC-like and tumor-initiating characteristics [60]. If the same mechanism acts in vivo (which is highly likely), this delineates EMT as a link coupling chronic inflammation in the HPV-infected human epithelium and malignant conversion of the latter.

It should be emphasized that HPV16 E6 and E7 oncogenes can promote EMT induction in human epithelial cells exposed to harmful external factors including carcinogenic ones. For example, EMT was shown to be induced in the HPV16 E6/E7-expressing cultures of human gingival keratinocytes and ectocervical cells exposed to ethanol [54,61] or cigarette smoke condensate [62], respectively. In the latter study, the cigarette smoke condensate-induced EMT in HPV16 E6/E7-expressing human ectocervical epithelial cells was associated with the activation of receptor tyrosine kinases that regulate TGFβ and other EMT-promoting players; the role of EMT in the initiation of HPV-positive cervical intraepithelial neoplasia (CIN) has been suggested by the researchers [62].

#### 2.1.3. HPV 16/18 Infection or Expression of HPV E6/E7 Genes Can Induce EMT in Non-Human Epithelial Cells

Notably, HPV16 and HPV18 infection and also HPV E6 and E7 oncogene products are able to induce EMT even in non-human mammalian epithelial cells. For instance, EMT markers were detected in the cervical epithelium of Balb/c mice infected with HPV16 and HPV18 [50]. Madin-Darby Canine Kidney (MDCK) cell line derived from the dog kidney epithelium, when stably expressing HPV16 E6 or E7, demonstrated a well-seen conversion from the epithelial (“cobblestone”-shaped) cell monolayer to mesenchymal (spindle-like) cell morphology [63]. Those morphological alterations in E6- and/or E7-expressing MDCK cells were accompanied by the expression and activation of the EMT-related transcriptional factors Twist, Slug, and ZEB1/2, which provided a molecular signature of EMT. Consistent with the acquisition of a mesenchymal phenotype, E6- and/or E7-expressing MDCK cells began to exhibit high migratory and invasive activities [63]. In a transgene mouse model, the carcinogenicity of βHPV8 E7 expression was confirmed [64]; in that study, the HPV8 E7 expression in basal keratinocytes of the murine epidermis was shown to induce EMT with the characteristic E/N cadherin exchange and acquired invasiveness. Mechanistically, the E7-conferred invasiveness of skin keratinocytes was explained by the fact that E7 somehow increases the levels of α3-integrin chain on the cell surface—this modulates the interactions of HPV8-positive keratinocytes with the ECM (first of all, with fibronectin) and thus alters their behavior toward the invasive one. In another study with the same transgene mouse model, it has been established that HPV8 E7 affects the junctional bridging proteins β-catenin and ZO-1 in HPV8-infected skin keratinocytes, while the re-localization of those proteins commonly implies EMT [65]. The researchers have suggested that the βHPV-associated EMT in skin keratinocytes contributes to the development of skin carcinomas [64,65]. All these data, obtained from non-human epithelial cell cultures and murine models, support the suggested ability of HPV to induce EMT in the infected epithelium.

#### 2.1.4. Induction of EMT in Human Epithelial Cells Infected by Low-Risk HPV or Treated with E7-Containing Exosomes

Interestingly, overexpression of even the low-risk HPV11 subtype E6/E7 in human nasal epithelial hyperplasia (HNEpC)-derived cells induced EMT in them, which was manifested by an increase in N-cadherin and a decrease in E-cadherin, as well as an enhancement in the cell migratory capacity [66]. The authors linked those effects to HPV11 E6/E7-enhanced activation of the JAK2/STAT3 pathway and suggested that such an EMT-stimulating mechanism may sometimes result in the malignization of HNEpC [66].

It is worth noting that HPV E7 proteins, even when entering in the form of exosome cargo, are capable of inducing EMT in recipient epithelial cells. Kannan et al. [67] have shown that HPV E7-containing exosomes derived from HPV16-positive oropharyngeal cancer cells induced EMT in cultured human non-tumorigenic mammary epithelial cells (MCF-10A line). Those exosome-treated MCF-10A cells became HPV16 E7-harboring and exhibited an EMT-related phenotype that was manifested in the increased vimentin/E-cadherin expression ratio and their acquired capacity for invasion.

#### 2.1.5. Implication of HPV E5 in the EMT Induction in HPV-Infected Human Epithelial Cells

Besides HPV oncoproteins E6 and E7, another HPV oncogene product, namely E5, appears to be involved in the implementation of the EMT program in HPV-infected human epithelial cells. In an in vitro model with HPV16 E5-transfected human keratinocytes, the E5 expression-induced EMT was found to be due to the down-regulation of FGF receptor 2b (FGFR2b) along with the abnormal up-regulation of the mesenchymal FGFR2c isoform as a result of the switch in alternative splicing [68]. That splicing switch-based exchange in the FGFR2 isoforms altered the receptor specificity for its ligands (FGFs), thus triggering EMT via stimulation FGF/FGFR2c signaling pathway. The experiments with silencing HPV16 E5 or FGFR2 in the transfected keratinocytes and also with inhibition of their FGFR2 activity have confirmed the direct implication of oncoprotein E5 in the induction of EMT by means of FGFR2 isoform switching [68].

Taken together, these published data delineate the contribution of HPV E6, E7 and E5 (along with the loss of E2) to coupling EMT with processes of neoplastic transformation of HPV-infected epithelial cells.

Finalizing Section 2.1, it should be noted that for the reliable identification of EMT in vitro and in vivo, the detection of its molecular markers—such as the expression of vimentin, N-cadherin, etc.—must be supported by characteristic changes in cell morphology (e.g., the acquisition of a spindle-like or stellate shape, the loss of epithelial-type intercellular contacts) and cell behavior (primarily an increase in migratory capacity). Thus, the results of molecular analysis must be corroborated by in situ observations of changes in cell morphology and migratory activity.

### 2.2. EMT in HPV-Associated Intraepithelial Lesions In Vivo and Its Relationship with Precancerous Condition

Consistent with the above results of in vitro studies, data are accumulating on the EMT-stimulating effects of HPV infection in the human epithelium in vivo. It is believed that the acquired ability to migrate in keratinocytes infected with HPV and undergone EMT allows them to move upward through the epidermis to release the viral particles to the outside. On the other hand, the EMT-driven lateral migration of HPV-infected keratinocytes into adjacent areas of the epidermis may promote viral persistence and expansion of the infection zone (Figure 1). It is of importance that in vivo, HPV-induced EMT in the infected epithelium appears to contribute to cancer development.

#### 2.2.1. EMT in HPV-Associated Cervical Neoplasms

High-risk HPV infection in the cervix initially results in low-grade squamous intraepithelial lesions (LSILs), often referred to as cervical intraepithelial neoplasia grade 1 (CIN1). Such lesions, which are loci of viral replication, exhibit only slight signs of dysplasia; they are usually eliminated by the host immune system within one year. Otherwise, if LSILs persist, they may progress into high-grade squamous intraepithelial lesions (HSILs), also referred to as CIN2 (moderate dysplasia) or CIN3 (severe dysplasia and carcinoma in situ). When patients with HSILs in the cervix remain untreated, the risk of cervical cancer development is sharply escalated (reviewed in [69]).

An indirect indication of a potential relationship between EMT and progression from CIN to cancer was a publication about the reduced expression of miR-34a in the normal cervical epithelium and CIN infected with high-risk HPV compared with the HPV-negative ones; moreover, miR-34a expression levels were significantly lower in the samples of CIN and cervical cancer compared with normal cervical epithelium, as well as in CIN2 and CIN3 compared with CIN1 [70]. Although EMT markers were not studied by those researchers, the reduced expression miR-34a in HPV-infected epithelial cells suggests Snail activation and Snail-induced EMT [57], which may correlate with CIN progression.

Liu et al. [71] found elevated levels of Twist2 protein (one of the drivers of EMT) in the cervical tissue samples from patients with CIN and cervical carcinomas. Later, it was shown that the expression of N-cadherin and Notch (one of the key components of EMT-related signaling) was increased in the tissue samples of CIN and cervical cancer [72].

Two review articles from 2021 and 2023 summarized data available at the time that allowed the authors to present the high-risk HPV infection in the cervix, induction of EMT in cervical epithelium, formation of pre-malignant cervical lesions, and development of cervical cancer as a sequence of interconnected events driven by a number of HPV-associated carcinogenic and cancer-promoting factors [73,74]. A later review [75] also summarizes the in vivo data on the direct link between the severity of HPV infection in the cervix and the eventual development of cervical carcinomas, emphasizing the role of TGF-β1 and its interaction with HPV oncoproteins: this interaction leads to an increase in the TGF-β1 level in the TME, so that TGF-β1 triggers intratumoral EMT, thereby promoting malignant growth via the enhancement of angiogenesis and immunosuppression. Results of a recent study on a murine model [76] support the idea that the development of HPV-associated cervicovaginal carcinosarcomas is an EMT-driven process.

#### 2.2.2. Can EMT Markers Be a Prognostic Factor for Assessing the Risk of Developing Cancer?

There is an intriguing question: is it possible to use the detectable EMT markers in order to diagnose HPV-induced lesions and/or predict their potential for oncogenesis? It was reported in an early publication [77] that the downregulation of E-cadherin expression is closely associated with the progression of CIN, but not with HPV infection. Studying cervical tissue samples, Pang et al. [78] revealed positive correlations with Snail expression and inverse correlations with E-cadherin expression during the progression from cervicitis (cervical inflammation) to CINs and then to cervical carcinomas. This suggests that the detection of EMT markers may be considered a diagnostic and predictive parameter toward the development of cervical precancerous conditions and cervical cancers. In another study [79], probing biopsy samples during the progression of HPV-positive CIN disease has revealed a progressive decrease in the expression of E-cadherin and β-catenin, while the expression of vimentin was found to increase in CIN3 (the advanced stage of CIN) and particularly in invasive cervical carcinomas, which suggests a direct interrelation between HPV infection, the execution of the EMT scenario, and the evolution of CIN disease to cervical malignancies. In this study [79], the authors have noticed a potential significance of the detection of EMT markers to evaluate the risk of progression of CIN with subsequent development of cervical cancer.

In a review of 2023, Amin et al. [80] have tried to compile a set of molecular markers for predicting the progression and prognosis of HPV-induced cervical lesions to cervical cancer. From the reviewed data [80], it follows that in addition to detectable amounts of oxidative DNA modifications, the predictive markers may be miRNA-377 and the proteins p16^INK4a^, PD-L1, and claudins that are dysregulated within the high-risk lesions. It should be noted here that both miRNA-377 [81] and PD-L1 and claudins (see Section 1.2) are involved in EMT. Based on this, one can assume that some of the EMT markers may also be considered as potential predictive markers for the prognosis of HPV-induced cervical lesions; this assumption certainly needs to be tested.

Other researchers have proposed determining EMT markers (vimentin, Twist) in cervical epithelial cell samples for the early diagnosis of cervical lesions in both HPV-positive patients and HPV-negative patients, as the number of cervical cells expressing the EMT markers was found to correlate with the presence of lesions but not with HPV status [82]. In principle, these findings are consistent with the above results on the decrease in E-cadherin expression [77]. The fact that the EMT markers correlated with cervical lesions in HPV-negative cases as well [77,82] does not refute the suggested causal relationship between HPV infection, EMT, and cervical lesions (including precancerous ones) in HPV-positive patients. Perhaps, the detectable EMT signs are such a significant diagnostic factor for cervical lesions that it works regardless of whether the EMT was caused by HPV infection or by any other triggers (e.g., by HPV-free local inflammations) in HPV-negative lesions. In any event, the suggested possibility of using the EMT molecular signatures for diagnosis/prediction of HPV-induced lesions and their development into cancer remains to be examined in further clinical studies.

After revealing the implication of HPV16 E5 and FGFR2c in the EMT induction in cultured human keratinocytes [68], Ranieri et al. [83] found an analogous HPV16 E5-dependent and FGFR2c-involving mechanism of EMT in ectocervical LSILs. Under studying of the tissue LSIL samples, the positive correlation between the expression levels of E5 and FGFR2c was demonstrated, whereas the E5 expression inversely correlated with that of FGFR2b (an epithelial isoform) and cytokeratin 10 (K10, an epithelial marker). Furthermore, HPV16 E5 expression in LSIL samples was shown to positively correlate or overlap with the expression of EMT-associated transcription factors Snail1, Snail2, and ZEB1 [83], implying the ability of E5 to initiate or promote EMT at LSIL loci. In support of those results obtained on tissue LSIL samples, the down-regulation of E5 in human cervical LSIL-derived cells (W12), containing episomal HPV16, by siRNA yielded the repression of FGFR2c, Snail1, Snail2, and ZEB1 along with re-expression of FGFR1, FGFR2b and K10, which indicates a recovery of the epithelial phenotype [83].

The HPV E5-dependent and FGFR2c-involving EMT appears to be quite universal, since after being revealed first in human gingival keratinocytes [68] and then in ectocervical LSILs [83], a similar mechanism was identified in precancerous lesions of the human anal epithelium [84]. Indeed, the positive correlations were found between the expression levels of HPV16 E5, FGFR2c, and EMT-related transcription factors (Snail1, Snail2 and ZEB1) in a study of samples of HPV-positive anal intraepithelial lesions [84].

The results from meta-transcriptomic studies of anal biopsies with LSILs or HSILs, as well as anal squamous cell carcinomas (SCCs) have shown that microRNAs responsively expressed in the HPV-infected anal epithelium may target a number of cancer-promoting genes including the EMT-related ones [85]; the researchers suggested that even low-risk HPV types, such as HPV9, 10, and 14 enriched across the lesion grades, may still contribute to the progression from anal LSILs to anal SCCs.

#### 2.2.3. The Involvement of EMT in HPV-Associated Tumorigenesis May Include Inflammation, Immunodeficiency and Co-Infection with Other Viruses

**Figure 3:**
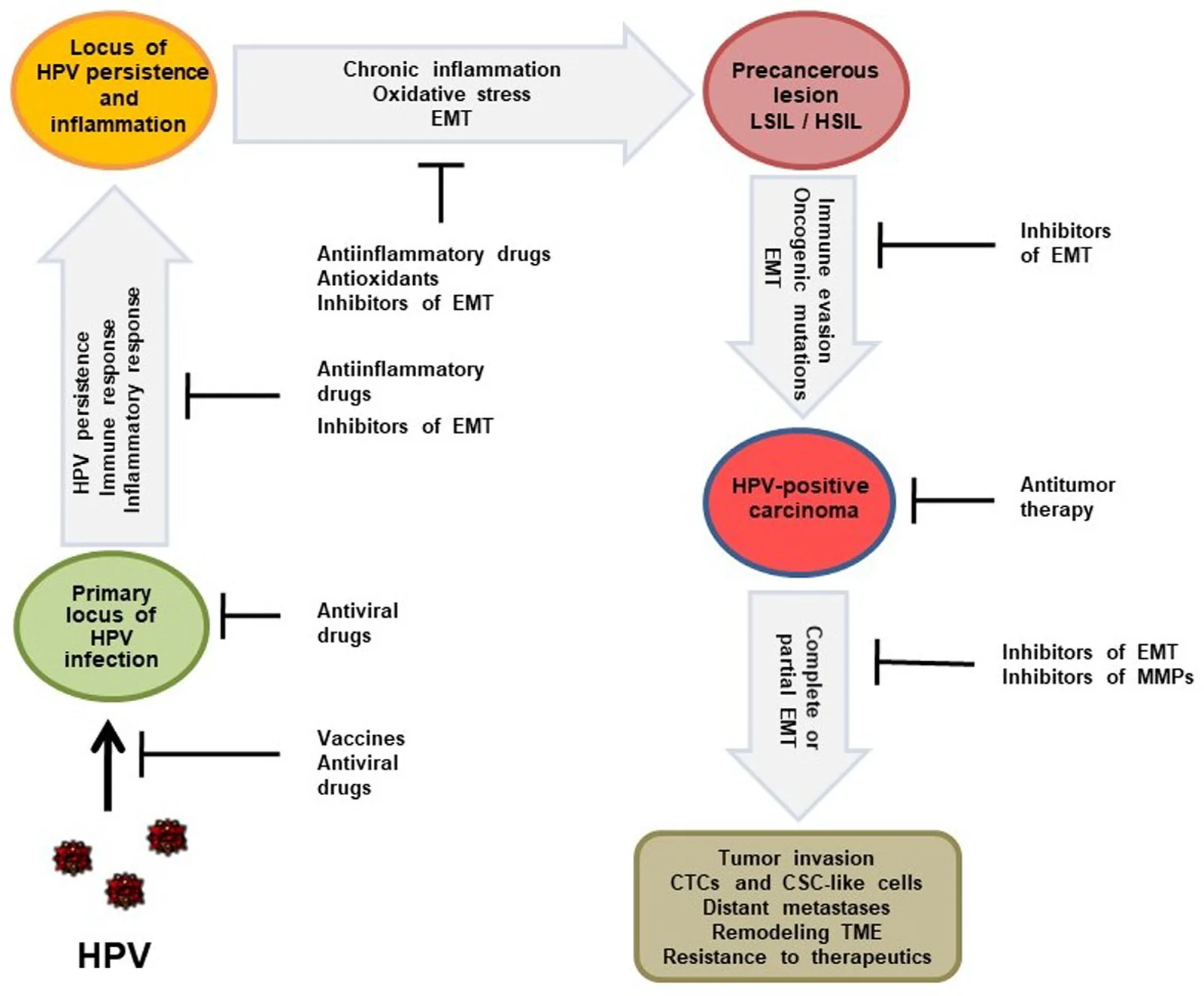
Hypothetical scheme illustrating the stepwise pathological progression from primary HPV infection to the development of HPV-positive carcinoma, as well as an indication of the involvement of EMT and potential ways of prophylactic or therapeutic intervention at each stage. Abbreviations: HPV—human papillomavirus; EMT—epithelial-to-mesenchymal transition; LSIL—Low-grade squamous intraepithelial lesion; HSIL—High-grade squamous intraepithelial lesion; MMPs—Matrix metalloproteinases.

Taking all the above data together, it can be concluded that (i) EMT occurs in HPV-associated human cervical and anal epithelial lesions, including precancerous ones; (ii) besides HPV E6 and E7, oncoprotein HPV E5 and the E5-mediated stimulation of FGF/FGFR2c-signaling may be the drivers in this process. Moreover, there appears to be a direct relationship between the intensity of EMT within HPV-infected human epithelium and the progression of pathological conditions in the affected tissue. Based on the data presented above, and taking into account the role of HPV-associated inflammation highlighted in the reviews [60,73,74], a hypothetical sequence of events can be outlined: HPV infection -> inflammation locus -> precancerous lesion -> carcinoma (see Figure 3). In some (exceptional) cases, the EMT-driven development of neoplasia in the HPV-infected epithelium may be accelerated by the administration of immunosuppressants [6] or co-infection with human immunodeficiency virus 1 (HIV-1) [86], or Epstein–Barr virus (EBV) [87,88].

#### 2.2.4. The Potential Involvement of EMT in Malignant Transformation of HPV-Associated Benign Neoplasms

A possibility of the malignant transformation of benign nasal neoplasms caused by papilloma viruses should also be considered in connection with EMT initiated in the virus-infected epithelial cells. The upregulated expression of an EMT-related gene set has been suggested as one of the drivers of malignant transformation of sinonasal inverted papilloma [89]. In HPV11-infected nasal mucosal epithelial hyperplasia, its malignant progression may be initiated following the E6/E7-mediated activation of JAK2/STAT3 pathway which promotes EMT [66].

The situation with epidermodysplasia verruciformis (EV) is a separate issue. EV is a rare precancerous skin disease characterized by extreme susceptibility to certain HPVs (in particular, HPV5 and HPV8), yielding widespread warts with a high risk of developing cutaneous SCCs [6]. The malignant transformation of EV lesions appears to involve an EMT-like process, in which oncogenic HPV5 or HPV8 affects the stability of intercellular junctions and signaling in the infected keratinocytes, promoting tumorigenesis. Heuser et al. [65] found that the E7 oncoproteins of HPV5 and HPV8, which are typically abundant in EV lesions, cause the upregulation and relocalization of β-catenin and ZO-1 (the events frequently associated with EMT and tumor invasion) in the skin of EV patients. However, further detailed studies are needed to establish the links between HPV5/8 infection and EMT in the context of the development of EV lesions into skin cancer.

#### 2.2.5. The Potential Involvement of HPV-Associated EMT in the Development of Lung Cancer

Intriguingly, although it is not yet proven that human lung cancer can be induced by HPV, a comparative study has shown the increased expression of inflammatory cytokines and EMT markers (N-cadherin, Slug, Twist) in patients’ HPV-positive lung cancer samples as well as HPV-positive control lung tissue samples as compared with the respective HPV-negative samples [90]. Based on those findings, the researchers suggested that HPV infection in human lungs induces inflammation and EMT, which may contribute to the development of lung cancer [90]. Their suggestion is consistent with the hypothetical scheme presented in Figure 3, where HPV-induced inflammation may precede the precancerous lesion state and the subsequent development of HPV-positive carcinoma; however, more detailed in situ studies of human lung epithelium are needed to confirm this.

Thus, the detection of EMT in patients’ epithelial lesions may indicate pathological progression and a high risk of cancer development. However, the question of whether the detection of EMT markers in HPV-induced epithelial lesions can be used to assess cancer risk remains unresolved. One challenge is that, in biopsy samples, it is quite difficult to distinguish epithelial cells that have undergone EMT—and thus exhibit a mesenchymal phenotype—from genuine mesenchymal cells (such as fibroblasts, myofibroblasts, pericytes, macrophages, and others) that may be present and accumulate in HPV-infected lesions. Further research is needed to identify EMT-related markers that could serve as biomarkers of HPV-driven carcinogenesis.

### 2.3. How Does EMT Contribute to Malignant Transformation of HPV-Infected Epithelial Cells?

#### 2.3.1. Oncogenic Effects of HPV Infection or HPV E6, E7 and E7 Expression in Human Epithelial Cells

From the data presented in Section 2.1 and Section 2.2 it follows that the induction and implementation of the EMT scenario may be essential (if not necessary) steps for the malignant transformation of HPV-infected epithelial cells. In fact, EMT appears to be one of the critical events that promote the conversion of HPV-affected epithelial cells into malignant (tumor-initiating) cells. In the in vitro experiments with human keratinocytes immortalized by either HPV infection [47] or expression of HPV E6/7 oncoproteins [56], those cells did become tumorigenic only after the EMT induction and acquisition of a mesenchymal phenotype. It is of utmost importance to uncover the molecular mechanisms by which HPV-associated EMT contributes to the conversion of partially transformed epithelial cells into fully malignant cells that are capable of forming aggressive tumors with all hallmarks of cancer. Unfortunately, this issue is not yet clear, and at present, one can only put forward suggestions and discuss possible options.

It is well-known that infection of human epithelial cells with high-risk HPVs can exert carcinogenic effects that are similar to the consequences of oncogenic mutations. In particular, HPV oncoproteins E6 and E7 are able to compromise the onco-suppressor functions of p53 and pRb, thus contributing to tumorigenesis (reviewed in [57,91]).

Also, HPV oncoproteins E6 and E7 can upregulate the expression/activity of hTERT in HPV-infected cells (see for a review [92,93]. In parallel, the HPV integration-induced alterations in DNA (de)methylation, histone modifications, chromatin remodeling, and non-coding RNA patterns contribute to oncogenesis by passivating tumor suppressor genes, breaching transcriptional regulation, and resulting in genomic instability [94]. Apparently, all these HPV-induced effects may confer on the infected cells some of the characteristics associated with cancer cells, such as the ability to divide indefinitely (immortalization), constant autostimulation of their own proliferation, resistance to replicative senescence and some forms of apoptosis, and others.

Of note, high-risk HPV oncoproteins E6, E7 [69,95] and E5 [96] enable HPV-infected keratinocytes to evade the host immune response; this immune evasion leads to long-term persistence of the viral infection and promotes malignant transformation of the affected epithelium with subsequent tumorigenesis. Nevertheless, the established effects of high-risk HPVs on p53, pRb, hTERT, and epigenetic regulation in target cells, as well as the resistance of HPV-carrying keratinocytes to the host immune system, appear to be necessary, but not yet sufficient, for cancer development [91,92,93].

#### 2.3.2. Hypothetical Mechanisms of the Involvement of HPV-Associated EMT in Malignant Transformation of HPV-Infected Human Epithelial Cells

In this context, EMT may represent precisely that series of changes in the properties of a partially transformed (HPV-infected) epithelial cell that will make that cell truly malignant. HPV-associated EMT may confer cancer cell-like or even CSC-like properties in infected epithelial cells, with their capacity for anchorage-independent growth, invasiveness, and self-renewal [47,56,60,97], which appears to be a crucial step. Based on experimentally obtained data on the EMT induction in cultures of human keratinocytes exposed to external carcinogenic factors [54,61,62], it can be assumed that an epithelial cell, having undergone EMT and acquired (temporarily) a mesenchymal phenotype, has a much higher chance of transforming into a malignant compared to a cell that retains an epithelial phenotype under the same carcinogenic exposure. It seems likely that the EMT of an HPV-transformed cell into a CSC-like phenotype and its prolonged remaining in this state ensure the most effective implementation of the entire set of oncogenic factors, including the integration of high-risk HPVs. By the entire set of oncogenic factors here is meant that “fatal” combination of carcinogenic interventions into the genome, epigenetic changes, disturbances in ligand-receptor interactions, deregulation in the cell cycle and functioning of transcription factors and signaling pathways, etc. All of this combined creates a malignant cell that has escaped immunosurveillance and is capable of initiating tumor development. If this happens in vivo, HPV-infected human epithelial cells—by acquiring CSC properties through HPV-associated EMT—may transform into malignant cells that initiate tumor development and give rise to aggressive carcinomas.

On the other hand, an alternative or parallel mechanism may operate, when high-risk HPV infection in an epithelial stem cell (possibly, in combination with exposure to other carcinogenic agents or tumor promoters) transforms it into a CSC, while massive EMT may occur in neighboring epithelial cells as a result of the viral infection and/or other stimuli such as TGF-β, FGF, TNF, etc. In the case of massive EMT, the microenvironment of a newly emerged CSC and its progeny becomes enriched with a population of mobile cells with a mesenchymal phenotype that actively remodel the surrounding ECM and stimulate angiogenesis, thus creating a tumorigenic niche with immunosuppressive and cancer-promoting conditions [75]. Such HPV/EMT-induced alterations in the microenvironment of CSCs may facilitate and accelerate the local cancer cell evolution and selection of malignant clones, ultimately leading to tumor development and progression.

At the molecular level, the mechanism coupling HPV-induced EMT with HPV-initiated tumorigenesis seems complex and multilevel. In addition to such EMT-related transcription factors as Snail, Slug, Twist, and ZEB1/2, this mechanism may involve the transcription factor Six1 [47,49] and certain enzymes such as Nox1 [54] and hTERT [56]. Besides the ‘canonical’ EMT-promoting pathways such as Wnt/β-catenin-, Notch-, and Hedgehog-signaling, the HPV-induced activation of TGF-β-signaling [47,75] and/or FGF/FGFR2c-signaling [68,83,84] and/or TRPC3/Ca^2+^ signaling [50] appears to contribute to HPV-associated EMT in the human epithelium infected with the high-risk HPV subtypes (HPV16/18). Inflammatory mediators (in particular, TNFα [60]) may also play a role in the formation of HPV16/18-conferred EMT/CSC-like phenotype. In turn, the low-risk HPV11 subtype may initiate EMT (and afterwards even acquisition of tumorigenicity) in the infected nasal mucosal epithelial hyperplasia cells through the activation of JAK2/STAT3 pathway by E6/E7 [66].

Considering that, on the one hand, Nox1 and TNFα are known as inducers of endogenous oxidative stress with intracellular ROS generation, and on the other hand, Nox1 [54] and TNFα [60] have been shown to cause EMT in HPV-infected human epithelial cells, one can assume the contribution of excessive ROS to both HPV-associated EMT and HPV/EMT-driven malignant transformation of those cells. Indeed, it is not difficult to imagine such a sequence of events when HPV infection first provokes a local inflammation in the affected epithelium and then inflammation-induced ROS, acting together with HPV oncoproteins, trigger EMT and complete the malignant transformation of the involved cells. Endogenous oxidative stress is really induced in HPV-infected cells [98,99], while the generated ROS can be a cause of both oncogenic mutations and EMT that promotes carcinogenesis. The above-mentioned presence of oxidative DNA modifications [80] and also the reviewed links between ROS and the induction of EMT in HPV-positive cervical lesions [99] support the idea of a contribution of oxidative stress to HPV/EMT-driven tumorigenesis.

Such a diversity in the intracellular events, regulatory components and signaling pathways that are causally involved in HPV/EMT-driven tumorigenesis may mean that its mechanisms vary in different cases. But in any event, the HPV-stimulated tumorigenesis appears to be EMT-addicted.

### 2.4. How Can EMT-Associated Malignant Transformation of HPV-Infected Human Epithelium Be Prevented?

Clearly, the risk of most HPV-related cancers can be reduced with anti-HPV vaccines and regular hygiene and surveillance of the skin, mucous membranes, and ano-genital area. However, what to do, if successful vaccination has not been administered (or is impossible) and a positive (high-risk) HPV-status has already been detected? In this regard, the strategy of so-called chemoprevention (i.e., the prophylactic use of natural, synthetic, or biological agents to suppress or slow the development of cancer in high-risk individuals) looks entirely rational. Moreover, EMT may be a druggable target for breaking the sequence of events leading to the progression from HPV infection to cancer (see Figure 3). In terms of the chemoprevention of HPV-associated cancer, the use of EMT inhibitors alone or in combination with anti-inflammatory drugs appears to be a possible way to prevent the fatal progression from primary HPV-induced lesions to carcinomas. The question is, which inhibitor(s) might be suitable for this?

The previous Section 2.1, Section 2.2 and Section 2.3 presented several key factors, molecular axes and signaling pathways that seem critical in the mechanism of HPV-associated EMT that leads to the development of cancers. Consequently, some of those EMT-related factors, axes and pathways may be considered as potential targets and their inhibitors may be used for the chemoprevention of HPV-associated tumorigenesis. For example, pyr3, a selective small molecule inhibitor of TRPC3, was shown to effectively suppress EMT in HPV 16/18-infected human cervical epithelial cell cultures and also in epithelium of HPV 16/18-infected mice [50]. In another study [55], EMT and EMT-conferred invasiveness in HPV16 E6/E7-expressing human skin fibroblasts were suppressed by dasatinib (a tyrosine kinase inhibitor). Although pyr3 has no clinical application, while dasatinib is an approved anticancer drug, its administration before diagnosed malignancy is inapplicable; similar, in vitro models may be fruitfully used for screening and testing various compounds to find a suitable small molecule agent to inhibit the cancer development-promoting EMT in HPV-infected epithelial areas.

In modern medicine there is a demand for the development and production of pharmacological EMT inhibitors to combat fibrotic disorders (e.g., pulmonary fibrosis and kidney or liver fibrosis) and, especially, to combat tumor invasion, metastasis spread and drug resistance in cancers. Therefore, pharmacologists are devoting significant efforts to finding or creating and testing certain compounds that can inhibit EMT in normal epithelial cells and carcinoma cells; a number of promising agents are currently at the stage of preclinical and clinical trials (see for a review [100,101,102]).

Taking into consideration that TGF-β is the master inducer of EMT [75], one can suppose that selective blockers or antagonists of TGF-β and its receptors seem usable for inhibiting EMT in HPV-infected human epithelium in order to prevent its malignant transformation. Besides TGF-β receptors, downstream molecular targets may be appropriate as well. At present, various inhibitors targeting TGF-β ligand/receptor are produced, tested, and promoted as inhibitors of EMT and potential remedies against fibrosis or cancer (reviewed in [103,104]). It seems possible that some of those drugs would be effective in terms of the chemoprevention of HPV-associated cancers.

In turn, the significance of the FGF/FGFRc2 pathway has been demonstrated for HPV-associated EMT in human gingival keratinocytes [68], ectocervical epithelium [83] and anal epithelium [84]; therefore, inhibitors targeting this pathway may prevent the HPV-provoked development of oral, cervical and anal carcinomas. Inhibitors of other EMT-related pathways (Wnt/β-catenin, Notch, Hedgehog, etc.) also deserve attention.

Suitable inhibitors of HPV-induced EMT may be chosen from natural products or their derivatives such as carotenoids and retinoids, flavonoids and polyphenols, and some other phytochemicals. A large range of natural products/derivatives somehow exerting inhibitory and/or reversing effects on EMT has been considered in topical reviews [105,106]. This phytochemical-based approach appears promising, as most beneficial carotenoids, flavonoids, and polyphenols (e.g., lycopene, resveratrol, curcumin, EGCG, and others) are nontoxic components of food and possess anti-inflammatory and antioxidant properties, while attenuation of inflammation and oxidative stress is thought to be a correct way to reduce the risk of cancer. If the “natural” inhibitors of EMT in parallel act as anti-inflammatory agents and ROS scavengers, such compounds may become effective and multitarget tools in the prevention of HPV-associated cancers. However, there is currently a lack of evidence that EMT in HPV-infected human epithelium can be prevented or reversed using the aforementioned plant-derived substances. Clearly, additional studies using appropriate (HPV-related) models are required to investigate this possibility.

Obviously, EMT inhibitors should be used with caution in the cases of advanced lesions with ulcers and tissue erosion. Since EMT normally contributes to wound healing and tissue regeneration (see Figure 2 and [16,17]), potent EMT suppression in severely damaged areas may abolish EMT-dependent healing/regeneration of erosive lesions. Nevertheless, the idea of using appropriate EMT inhibitors to prevent the development of HPV-associated cancers (see Figure 3) seems attractive and warrants verification in preclinical and clinical studies.

## 3. HPV-Associated EMT in Malignant Tumors of Different Locations

Just as cells of normal epithelium undergo EMT, carcinoma cells can also change their epithelial phenotype to a mesenchymal and CSC-like phenotype, and this reversible process significantly contributes to cancer pathogenesis by driving tumor invasion and metastasis spread (see Section 1.2 and Figure 2). This section reviews the peculiarities of EMT pathway in different HPV-positive cancers along with potential modalities to beneficially target the HPV-associated EMT in terms of antitumor therapy.

### 3.1. Cervical Cancers

Carcinomas of the uterine cervix are the type of HPV-infected malignancies in which the machinery and consequences of EMT have been explored particularly thoroughly. The availability of cell lines derived from HPV-infected or HPV-uninfected cervical carcinomas has made it possible to study the molecular mechanisms of stimulation and regulation of HPV-associated EMT in human cervical tumors. There are cervical cancer cell lines natively infected with HPV16 (SiHa, CaSki and HCC-94 lines) or HPV18 (HeLa line), while HPV-free cervical cancer cell lines (e.g., Cx line or C33A line) can be used as negative controls or experimental tools for ectopic overexpression of specific HPV proteins.

#### 3.1.1. EMT-Related Effects in Human Cervical Cancer Cells Being HPV-Positive or Ectopically Expressing HPV Proteins

Even in early studies on HPVs and cervical cancer, the phenomenon of EMT and its molecular markers were revealed in cancer cell cultures derived from HPV16-positive cervical tumors, as well as in the tissue samples of HPV16-positive cervical tumors [71,107,108,109,110]. Molecular signatures of EMT, such as decreased E-cadherin levels and expression of vimentin, N-cadherin and αSMA were detected in FGF2/4-stimulated CxWJ cells ectopically expressing HPV16 E6/E7 and exhibiting invasive capacity [107]. Shostak et al. [108] have shown that HPV16 positivity in CaSki cells is responsible for the enhanced expression of KIAA1199 (often referred to as CEPIM: cell migration-inducing protein), while the latter promotes EGF-induced EMT. Such KIAA1199-mediated EMT in EGF-stimulated CaSki cells was manifested in the decreased expression and re-localization of E-cadherin and ZO-1 along with the expression of N-cadherin, vimentin, Slug and fibronectin that altogether were associated with the migratory capacity and invasiveness of the HPV16-positive cervical cancer cells [108]. In turn, HPV16-positive SiHa cells exhibited the upregulated expression of Twist2 and vimentin along with the downregulated expression of E-cadherin; those EMT-driven alterations in the protein expression in the cells were associated with their mesenchymal-like morphology and invasive capacity [71]. In another study [109], the decreased E-cadherin expression and increased P-cadherin expression were found to correlate with HPV16 E6/E7 levels in high-grade cervical cancer lesions as well as in SiHa and CaSki cells, which enhanced the cell migration and invasion.

#### 3.1.2. Protein Factors Influencing EMT in HPV-Positive Human Cervical Cancer Cells

Using siRNA-based silencing, it was demonstrated that it is HPV16 E6 that is responsible for the expression of Slug and Twist under EMT in SiHa cells [110]. In a later study, experiments with HPV16 E6 silencing or overexpression in SiHa and C33A cells, respectively, confirmed the key role of E6 in EMT-mediated invasion and migration of cervical cancer cells as well as in maintaining the cancer stemness characteristics [111]. The siRNA-caused knockdown of DLL4 (Delta-like ligand 4, a regulator of Notch signaling) in SiHa and CaSki cells was found to reverse EMT in those HPV16-positive cervical cancer cell lines, which was manifested in the upregulation of E-cadherin and downregulation of vimentin, MMP2, and MMP9, as well as the suppression of cell migration and invasion [112]. The data from other experiments with SiHa cells indicated that HPV16 E6 and E7 upregulate the expression of PiwiL1, which mediates the enhanced expression of EMT-related genes regulating cancer cell migration [59]. The recently revealed HPV16 E7-APC2-SPIN4 axis was shown to be responsible for the stimulation of Wnt/β-catenin pathway followed by the enhancement of cancer stemness and migratory/invasive capability in cells of human cervical carcinoma cell lines (CaSki and SiHa) [113].

Comparing studies on cell cultures and xenografts with in situ probing of tumor tissue samples has enabled to identify a number of various molecular factors causally involved in the initiation, implementation, and regulation of EMT in HPV-related cervical cancers. Among the cellular genes and their protein products that promote EMT in HPV-positive cervical tumor cells, the following have been identified: PiwiL1, a protein regulating stem cell renewal and RNA silencing [59]; forkhead box protein C2 (FOXC2) [72]; Y box-binding protein 1 (YB-1) [78]; KIAA1199 (CEMIP) [108]; delta-like ligand 4 (DLL4) [112]; URI (the unconventional prefoldin RPB5 interactor) [114]; claudin-1 (CLDN1) [115]; cAMP-responsive element-binding protein 1 (CREB1) [116]; homebox (HOX) cluster gene products HOXA10 and HOXB13 [117]; gelsolin (a protein regulating actin filament dynamics) [118,119]; lysine-specific demethylase 1 (LSD1) [120]; *PIR* gene and its protein product pirin [121,122]; Non-SMC Condensin I Complex Subunit H (NCAPH) [123]; Ras-associated binding protein Rab31 and mitogen-activated protein kinase 6 (MAPK6) [124]; kinesin family member 2A (KIF2A, an ATP-driven microtubule-associated protein) [125]; methyltransferase like protein (METTL14), insulin like growth factor 2 mRNA-binding protein 3 (IGF2BP3) and fascin actin-bundling protein 1 (FSCN1) [126], transmembrane protein 45A (TMEM45A) [127], and MSX1, a transcription factor of the homeobox family [128]. The upregulated expression and activation of all those EMT-promoting proteins were shown to be mediated by HPV E6/E7. Therefore, there are multiple HPV-responsive mechanisms by which the HPV oncoproteins E6 and E7 can induce and promote EMT in cervical cancer cells.

#### 3.1.3. Non-Coding RNAs Influencing EMT in HPV-Positive Human Cervical Cancer Cells

In addition to the above protein regulators, epigenetic factors such as microRNAs (miR), long non-coding RNAs and circular RNAs are known to influence the EMT program in cervical tumors. Reduced expression of miR-34a (a suppressor of Snail) was detected in cervical cancer tissue samples [70], which may lead to intratumoral Snail activation and Snail-mediated EMT [57]. In turn, the overexpression of miR-218 was revealed to inhibit EMT, migration and invasiveness in cultured cervical cancer cells, while HPV16 E6 was able to downregulate miR-218 in them, thereby facilitating EMT and invasion in HPV16-positive cervical carcinomas [129]. In another study, HPV E6 was found to upregulate miR-20b in cervical carcinomas and also in HPV-positive cancer cell lines HeLa, SiHa and CaSki which resulted in both EMT and enhanced migration/invasion in the miR-20b-enriched cervical cancer cells [130]. Mechanistically, miR-20b-conferred enhancement of invasion of HPV-positive cervical carcinoma cells has been explained by the ability of miR-20b to suppress tissue inhibitor of metalloproteinase 2 (TIMP-2) via the suggested HPV/miR-20b/TIMP-2 pathway [130]. The effects of either microRNA on HPV-associated EMT in human cervical cancers may depend on tumor histotype and HPV type: for example, miR-9-5p was shown to downregulate Twist1 (a transcription factor involved in EMT) in the HPV18-positive cervical adenocarcinoma-derived HeLa cell line, but not in the SiHa cell line and the CaSki cell line (both derived from HPV16-positive cervical SCCs) [131].

It has been demonstrated that miR-21 is induced in HPV16-positive SiHa and HPV18-positive HeLa cervical cancer cells, which may promote EMT through the TGFβ R2/hTERC pathway [132]. According to recently reviewed data [133], miR-21 is frequently upregulated in both HPV-positive cervical cancer cell lines and HPV-positive cervical cancer tissue samples, where it has been found to inactivate tumor suppressors and promote intratumoral EMT with stromal remodeling. Importantly, artificially reducing miR-21 levels in human cervical cancer cells reverses EMT and may have a therapeutic effect [133]. In turn, the knockdown of E6, E7 in HPV16-positive SiHa and CaSki cells was shown to result in the upregulation of miR-23b-3p expression, which was associated with the inhibition of ICAT (an inhibitor of β-catenin and T-cell factor) expression and suppression of EMT, migration, and invasiveness in those cells [134]. Based on their finding that ICAT is a target gene of miR-23b-3p and is negatively regulated by this microRNA, the researchers suggested the HPV16 E6, E7/miR-23b-3p/ICAT axis as one of the promoters of EMT and EMT-mediated invasiveness in HPV16-positive human cervical tumors [134].

Regarding representatives of other RNA classes, a review article of 2018 considered and discussed both the HPV-associated expression of a number of long non-coding RNAs and their involvement in modulating pathways that drive EMT, such as PI3K/Akt/mTOR, Wnt/β-catenin, and Notch [135]. Later, Lai et al. [136] have shown that in cervical SSCs, HPV16 E6 and E7 upregulated the expression of long non-coding RNA SNHG12 through the modulation of transcription factor c-Myc, while excess SNHG12 facilitated EMT through ERK/Slug/E-cadherin pathway. As a result, the HPV16-infected cervical carcinoma cells became SNHG12-enriched and exhibited the EMT-conferred phenotype and characteristics including enhanced migration and invasion [136].

Wang et al. [137] reported that the expression of circular RNA circ_PVT1 is upregulated in human cervical carcinomas and also in cervical carcinoma cell lines including both HPV-negative (C33A) and HPV-positive (CaSki, HCC-94 and HeLa) cell lines. The increase in the intracellular circRNA_PVT1 level correlated with the EMT induction and enhancement of cancer cell migration, tumor invasion, and pulmonary metastases [137]; however, the interactions between circRNA_PVT1 and HPV remain to be established. Further focused studies will likely identify other members of the non-coding RNA family that somehow influence the EMT in HPV-positive cervical carcinomas.

**Figure 4:**
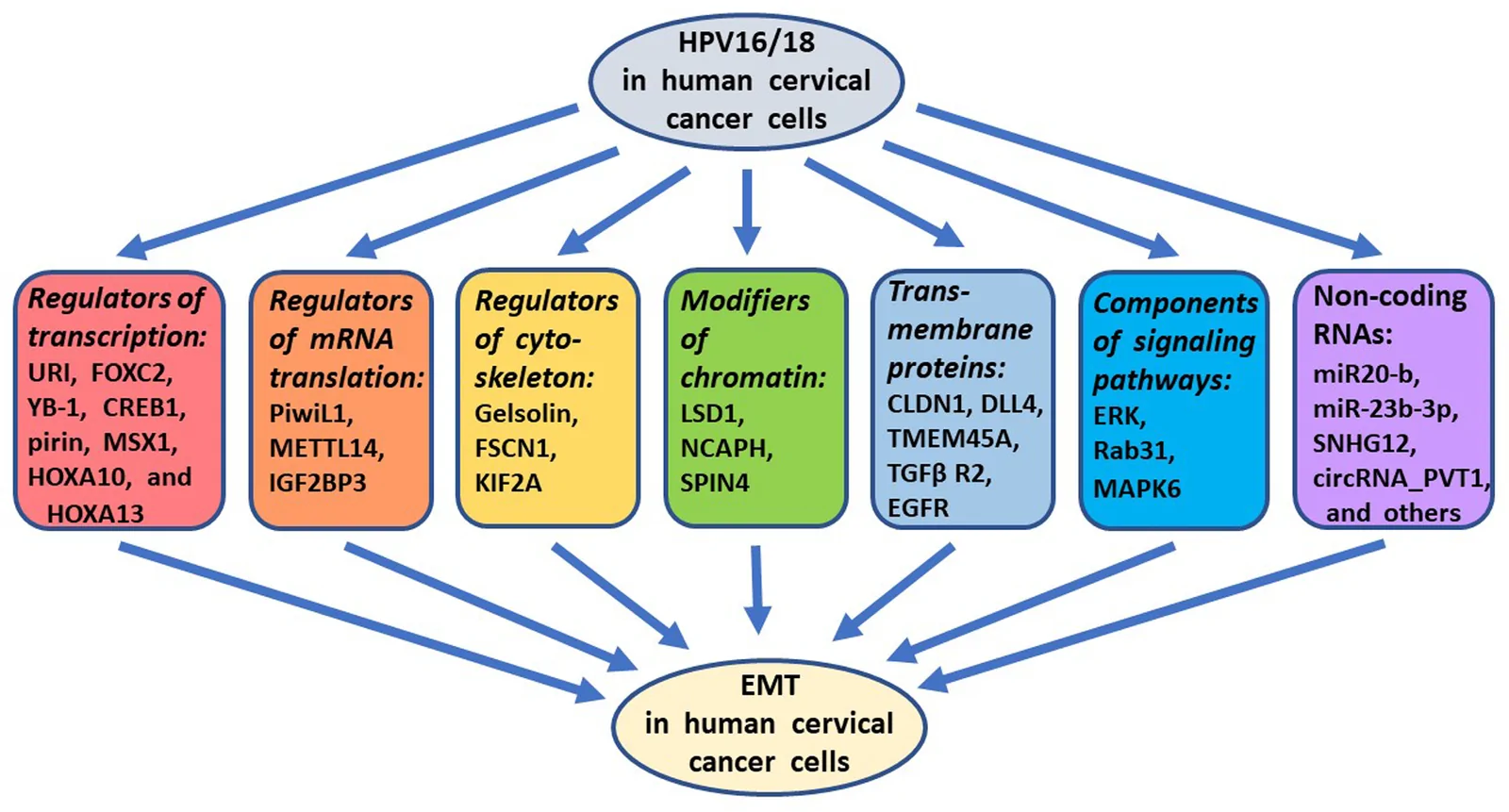
Simplified illustration showing distinct groups of key cellular regulators that are involved in the molecular crosstalk between HPV16/18 and the process of EMT in human cervical cancer cells.

It is impossible not to note the large number of diverse endogenous factors involved in the molecular crosstalk between HPV and the process of EMT in human cervical cancer cells; these factors can be divided into approximately seven large groups (see Figure 4).

#### 3.1.4. Mechanistic Links Between HPV-Associated EMT and Its Biological Effects in HPV-Positive Human Cervical Cancer Cells

The above studies on identifying molecular factors involved in EMT in HPV-positive cervical carcinomas (see Figure 4) have helped to elucidate the machinery of some critical manifestations of the intratumoral EMT. In particular, the revealed interactions of HPV E6/E7 with Rab31 [124], gelsolin [118,119], KIF2A [125] and fascin [126] appear to define the rearrangement of cortical microfilaments and microtubules required for the formation of cellular ‘leading edge’ and lamellipodia that enable the directed movement of migrating cells, while it is cancer cell migration that underlies tumor invasion and metastases. Such an EMT promoter as KIAA1199 (CEMIP), whose expression is enhanced in HPV16-positive cervical cancer cells, not only contributes to the EMT phenotype marker switching via the activation of EGFR-related signaling [108] but also, being secreted, KIAA1199 depolymerizes extracellular hyaluronic acid [138], which can facilitate tumor cell migration through the ECM. Also, in the context of HPV-associated EMT, the identified HPV/miR-20b/TIMP-2 axis in human cervical carcinomas [130] explains how HPV-positive cancer cells may exhibit increased metalloproteinase 2 activity to degrade the ECM along their migration path, thus carrying out invasion and metastases. All those findings virtually delineate a set of potential molecular targets for combating the invasive and metastatic expansion of HPV-positive tumors.

Considering that angiogenesis and tumor vascularization are among the hallmarks of cancer that contribute to malignant growth, the role of HPV and EMT in the phenomenon of vasculogenic mimicry in cervical cancers should be mentioned here. Avila et al. [139] have reviewed how EMT, hypoxia, and activation of STAT3 and PI3K/AKT pathways are implicated in vasculogenic mimicry occurring in cervical tumors associated with chronic high-risk HPV infection. The data show that such EMT-promoting proteins as TXNDC5 and CHI3L1 are among the drivers of vasculogenic mimicry in high-risk HPV-infected cervical cancers, while erythropoietin, microRNAs miR-124 and miR-29b act as suppressors of this EMT-mediated pseudovascularization [139].

Interestingly, siRNA-induced knockdown of E-cadherin in primary cultures of cervical cancer cells was shown to confer a transient CSC-like phenotype and drug resistance [140]. Given that similar downregulation of E-cadherin occurs during EMT in both HPV-positive and HPV-negative cervical cancer cells (see above), the EMT-associated decrease in E-cadherin level may be a crucial event ensuring the emergence of CSC-like cells in cervical carcinomas, thus linking intratumoral EMT, cancer stemness and drug resistance.

#### 3.1.5. Can EMT Markers Be a Prognostic Factor for Human Cervical Cancer?

Although HPV positivity at diagnosis of cervical cancer predicts a more favorable outcome [4], the intratumoral EMT may aggravate the pathogenesis through invasion and metastases. For example, despite the fact that HPV-positive cervical cancers exhibit encouraging sensitivity to radiation therapy [12], the beneficial effect may only be limited to the primary tumor receiving local irradiation, while HPV-induced EMT may lead to distant metastases, which will reduce the effectiveness of radiation therapy.

The questions are: how the presence and intensity of EMT can be detected and assessed in biopsy specimens from patients with cervical cancer, and how such data should be taken into consideration in prognosis and treatment strategy? In principle, both HPV oncoproteins and typical molecular markers of EMT can be detected by the presence of their mRNAs in cervical cancer biopsies or scrapings. There are several publications that have already suggested or demonstrated the prognostic significance of EMT detection for cervical cancer [13,73,82,141].

It is possible that detection of EMT or its consequences in tumor biopsies or scrapings may predict the response of HPV-positive cervical carcinomas to chemotherapy and radiation therapy. In particular, one of the critical consequences of intratumoral EMT is the emergence of CSC-like cells exhibiting chemo- and radioresistance that can be the cause of tumor recurrence after treatment (see Section 1.2). Javed et al. [142] found that EMT and a high percentage of CD133+ cervical CSC-like cells in human invasive cervical cancer biopsies correlated with high tumor radioresistance. Meanwhile, the radiation response of human cervical CSCs was shown to depend on the initial proportion of cervical CD44+ CSCs and the physical state of HPV DNA [143]. Given that EMT-generated CSC-like cells are commonly chemoresistant, the EMT-based prognosis may also include predictions toward the responses of HPV-positive cervical carcinomas to some chemotherapeutics. Of course, further research is required taking into account such parameters as the patient’s age, tumor histotype, HPV type and HPV integration profile, etc., in order to accept EMT as a predictive marker for prognosis in HPV-positive cervical cancers.

A separate question concerns which specific EMT markers (or their combinations) are suitable for detecting EMT in tumor biopsy samples. It is important to note that elevated levels of EMT-associated proteins such as vimentin and N-cadherin in tumor tissue samples may result not from EMT, but rather from the intratumoral accumulation of non-cancerous mesenchymal cells (e.g., CAFs, vascular cells, immune cells, etc.). Therefore, it is necessary to develop methods and test systems with cell typing to distinguish carcinoma cells that have undergone EMT and genuine mesenchymal cells in tissue samples.

#### 3.1.6. Approaches to Inhibiting EMT in HPV-Positive Human Cervical Cancer Cells

Since the contribution of EMT to cancer pathogenesis is obvious, the efforts of many research groups are aimed at finding ways to inhibit the EMT process in target tumors. With regard to HPV-positive cervical cancer, Table 1 presents a number of preclinical studies in which successful attempts to inhibit EMT were made in cervical cancer-related models, along with the corresponding refs.

As follows from the data in Table 1, a number of the effective inhibitors of EMT in HPV-positive cervical cancer cells have already been identified in preclinical studies. These inhibitors include the nucleoside adenosine [145], Brazilian nematode antigens [146], plant-derived compounds [122,144,148,149,150,151,152], artificially prepared exosomes [153], FDA-approved drugs [147,154], anti-HPV E7 antibody-based affitoxins [155,156], and a blocker of Rho kinase [128]. The effects of adenosine [145] seem good; however, in those experiments, adenosine acted at fairly high concentrations (0.1–1 mM), which is difficult to achieve in vivo.

Although most of the data presented in Table 1 were obtained in vitro, at least three studies were performed on in vivo xenograft models [147,154,156], and their encouraging results provide hope that such approaches will be beneficial for patients. Furthermore, bazedoxifene [147] and bevacizumab [154] are known to be FDA-approved drugs, while bevacizumab is even recommended for advanced cervical cancer [157]. In turn, the recently constructed affitoxins ZHPV16E7-GrB [155] and ZHPV16-18E7-GrB [156] seem to be very promising agents that reverse EMT in human cervical cancer cells expressing E7 of HPV16 and/or HPV18; additionally, these agents are able to kill the target cancer cells via GrB-mediated mechanisms. The reported effects of plant-derived agents [122,144,148,149,150,151,152] are noteworthy as well, especially considering that curcumin and EGCG are already tested as potential drugs against cervical cancer in preclinical studies [158]. Perhaps some of those plant-derived inhibitors of EMT [122,144,148,149,150,151,152] could also be tested in studies aimed at finding ways to prevent the pathological progression from cervicitis to CIN and then to cervical carcinoma (see Section 2.4 and Figure 3).

Thus, the HPV-associated EMT in cervical tumors seems targetable and oncologists already have pharmacological tools to inhibit or reverse this process in vivo. The next task is to develop the optimal inhibitor combinations and protocols for their use to effectively target EMT in HPV-positive cervical tumors and improve treatment outcomes. In case of surgical intervention or ablation, EMT-inhibiting treatment should be used with caution, since such inhibitors may suppress EMT-dependent postoperative scar healing.

It should also be noted that, in parallel with inhibiting or reversing intratumoral EMT, its consequences must be addressed. By the time EMT inhibitors are administered, some cancer cells may have already undergone EMT and acquired chemoresistance, making them unresponsive to the inhibitors being used. In this regard, the use of agents that impede cancer cell migration, such as inhibitors of MMPs, may be beneficial (Figure 3).

### 3.2. Head and Neck Cancers

HPV, specifically strain 16, causes head and neck cancers, primarily in the oropharynx. The most typical HPV-positive head and neck cancer is thought to be squamous cell carcinoma (SCC); HPV-positive head and neck SCCs usually have a better prognosis than the HPV-negative ones [4]. In addition to HPV infection, tobacco smoking and alcohol consumption can be carcinogenic factors that cause HPV-negative oral SCC in humans.

Numerous findings indicate a significant contribution of intratumoral EMT to the development and pathogenesis of head and neck SCCs (reviewed in [159,160]); however, the relationship between the HPV status and EMT in such SCCs is not fully understood. Compared to the above situation with cervical cancer (Section 3.1), the data obtained in vitro on head and neck SCC-derived cell lines and in clinical studies of patients with head and neck SCCs do not enable to clearly determine how HPV is involved in EMT in human malignancies of such a type. Moreover, the published data seem quite contradictory: there are studies where EMT in cells of human oropharyngeal SCCs seems to be associated with the presence of HPV, whereas in other works either no relationship between HPV status and EMT was found, or even enhanced EMT was detected in the HPV-negative SCCs compared to the HPV-positive ones.

#### 3.2.1. Contribution of HPV Infection or HPV E6/E7 Overexpression to EMT in Cells of Human Head and Neck Carcinomas

For example, it was reported that HPV positivity in oropharyngeal SCCs was associated with a loss of the epithelial phenotype and induction of EMT, as well as with lymph node metastases [161,162]. According to those studies, prognoses and outcomes were paradoxically better in the HPV-positive SCC patient groups, despite the EMT-associated aggressive tumor cell phenotype that yields nodal metastases [161,162].

Being interrelated with intratumoral EMT induction [24,25], the expression of PD-L1 was shown to be upregulated and correlated with the expression of EMT markers (Twist 1, Snail) in the samples of HPV-positive tonsillar SCCs; all three parameters, namely HPV positivity, PD-L1 positivity, and detectable EMT were suggested as favorable prognostic factors [163]. The fact that HPV positivity in oropharyngeal SCCs may promote the intratumoral EMT has been demonstrated in vector-transduced SCC143/E7 cells overexpressing HPV16 E7: those E7 oncoprotein-enriched SCC cells, began to overexpress pirin (a protein product of PIR gene) that led to pirin-mediated EMT with E-/N-cadherin switch and enhanced cell migration as a result of EGFR/PI3K/Akt1/NRF2 signaling and PIR/NF-kB activation [164]. An analogous PIR-dependent mechanism of HPV16 E6/E7-stimulated EMT has previously been described for HPV16-positive cervical cancer cells [121] which suggests a common (PIR-involving) machinery in HPV16-driven EMT in both cervical and oropharyngeal carcinomas.

Immunocytochemical analysis of the samples from patients with oropharyngeal SCC has revealed that the levels of cell-membranous β-catenin were higher in HPV-positive tumors than in HPV-negative ones and increased in the case of regional metastases [165]. Such correlations between HPV presence, β-catenin expression and metastases indicate the EMT-inducing ability of HPV-positive SCCs; at the same time, no relationship between EMT markers and patient survival was found in that study [165]. Bioinformatic analysis has allowed Zhou et al. [166] to identify six genes (ITGA5, PLAU, PLAUR, SERPINE1, TGFB1 and VEGFC) that, when expressed, seem to be biomarkers of poor survival of patients with HPV-positive head and neck SCC; importantly, all of those genes are known to be involved in the EMT pathway in head and neck SCCs. According to Okuyama et al. [167], HPV can modulate the expression of transmembrane protein 16A (TMEM16A, a Ca^2+^-activated Cl^−^ channel) that forms a complex with EGF receptor and promotes EMT/metastases in HPV-positive head and neck cancer.

Proteomic and phospho-proteomic studies have revealed that the increased EMT potential in HPV-positive head and neck SCCs is associated with recurrence [168]. Another publication stated that the activation of PI3K/EMT pathway is one of the characteristic responses of HPV-positive oropharyngeal SCCs that are resistant to chemo-radiation treatment [169]; the researchers suggested a contribution of PI3K-mediated EMT to this chemo-radiation resistance.

Similarly to what happens in HPV-positive cervical cancers, pseudovascularization based on EMT-mediated vasculogenic mimicry takes place in HPV-infected oral SCCs as well [139]. Compared to the situation in HPV-positive tumors of the cervix, other molecular factors regulate vasculogenic mimicry in HPV-positive oral SCCs. In particular, HIF-1α, collagen XVI, LGR5, ALDH1, VE-cadherin and CD44 (a marker of CSCs and CSC-like cells) are promoters of EMT, whereas SOX7 is a suppressor of EMT and beclin-1 may ambivalently function as either a promoter or a suppressor of EMT under regulation of the process of vasculogenic mimicry in HPV-related oral SCCs [139].

There are recent publications indicating molecular mechanisms by which HPV stimulates partial EMT in oropharyngeal SCCs. Lim et al. [170] have described that splicing of HPV16 E6 promotes aggressive invasion in HPV-positive oropharyngeal SCC via endocytosis of E-cadherin. It has been revealed that a spliced isoform of HPV16 E6 (E6*I) induces endocytosis of E-cadherin from the cell membrane, thus causing partial EMT in HPV 16-positive SCC cells followed by activation of their invasion. This HPV16 E6 splicing-dependent mechanism may be blocked by introducing splice-switching oligonucleotides (SSOs) into the cells that inhibit the formation of E6*I and mitigate invasion [170]. It is noteworthy that in this case, partial EMT was implemented not by reducing the initial expression level of E-cadherin, but by its incomplete removal from the cell membrane via endocytosis; meanwhile, transcriptomic signatures of partial EMT in head and neck SCCs were found to correlate with poor patient survival [171].

In another recent study [172], the apolipoprotein B mRNA editing enzyme catalytic subunit 3B (APOBEC3B)/anti-silencing function 1B histone chaperone (ASF1B) axis is described as a molecular link between HPV status and TGF-β-driven EMT in human oral SCCs. It has been established that HPV infection substantially enhances APOBEC3B and ASF1B expression in oral SCC cells which is accompanied by the activation of TGF-β signaling and subsequent upregulation of EMT markers as well as increased invasiveness and migratory capacity in HPV-positive oral SCC cells as compared to the HPV-negative ones. The researchers characterize APOBEC3B/ASF1B axis as a pivotal pathway by which HPV infection activates TGF-β signaling and thus promotes EMT, while the latter contributes to the formation of aggressive lymphatic metastases [172].

Likewise, the critically altered epigenetic regulation appears to contribute to the EMT induction in HPV-positive head and neck cancers. In particular, hypermethylation of PRKCZ (a gene encoding protein kinase C-zeta) in human HPV-positive head and neck SCCs was shown to induce EMT and EMT-related invasion: HPV promotes the expression of DNA-methyltransferase 1 (DNMT1), which increases PRKCZ methylation, and then the methylation-mediated upregulation of PRKCZ product/activity leads to EMT via Cdc42-involving mechanism [173]. In a review of 2025 [94], the HPV integration-associated alterations in DNA methylation, histone modifications, chromatin remodeling, and non-coding RNA expression were considered as epigenetic triggers or promoters of EMT in human oral SCCs, which helps to understand the mechanistic link between HPV-induced EMT and epigenetic dysregulation in HPV-positive oral cancers.

According to recently published data [174], about of 20% of HPV-positive head and neck tumors had fibroblast-rich ecosystem architectures resembling HPV-negative tumors, and those tumors were associated with worse outcomes than other HPV-positive head and neck tumors at analogous stages. Co-culture experiments with cells derived from head and neck cancer patients revealed that the abundance of ECM-producing fibroblasts, CD8+ T cell dysfunction, and chemoresistant tumor cell phenotypes were associated with EMT-like tumor states [174]. Simkin et al. [175] reported that, when compared to HPV-negative head and neck SCCs, their HPV-positive counterparts exhibit reduced partial EMT with two distinct architectures: a partial EMT edge where it is associated with CAFs at the invasive front via TGFβ, and a partial EMT core in which tumor-infiltrating macrophages and neutrophils induce partial EMT via oncostatin M.

#### 3.2.2. EMT Can Occur in Cells of Human Head and Neck Carcinomas Regardless Their HPV Status or Even Be Suppressed in the Case of HPV Positivity

Although many publications indicate that HPV infection initiates or promotes EMT in head and neck SCCs [161,162,163,164,165,166,167,168,170,172,173,174], and specific molecular mechanisms have been described by which HPV contributes to EMT in those tumors [164,167,170,172,173], there are reports that EMT either was manifested regardless of HPV status [55,176,177,178] or even was more common (or more intensive) in HPV-negative head and neck SCCs than in the HPV-positive ones [9,10,175,179,180,181,182,183,184,185]. Such conflicting data show a dissimilarity with the situation in HPV-positive uterine tumors, where the presence of HPV clearly contributed to EMT (see Section 3.1), and also confuse the relationship between HPV and EMT in oropharyngeal SCCs. This discrepancy suggests at least two possible explanations: (i) unlike HPV-positive cervical cancers, oropharyngeal SCCs may be subject to stronger stimuli for EMT induction than HPV infection, and (ii) HPV-associated mechanisms exist that somehow suppress EMT in oropharyngeal SCCs.

In fact, several molecular mechanisms have been delineated that can inhibit EMT in HPV-positive head and neck SCCs. For example, parafibromin expression was found to positively correlate with HPV positivity and negatively correlate with EMT and cell migration/invasion in human head and neck SCCs [9]. Also, it was reported that HPV-positive head and neck SCCs have higher expression of SMAD4 compared to the HPV-negative ones, while the downregulation of SMAD4 in CCS cells is associated with EMT induction and resistance to cetuximab—exactly what was observed in HPV-negative head and neck SCCs [10]. Fontan et al. [58] found an inverse correlation between HPV E2 expression and Twist1 expression in HPV E2-positive head and neck cancer cell lines, suggesting that E2 represses Twist1 expression, thereby inhibiting EMT in HPV E2-positive head and neck SCCs and thus improving the outcome of patients with such tumors.

Other researchers [181,182] described that Krüppel-like factor 4 (KLF4, a zinc-finger transcription factor) inhibits EMT in human head and neck SCCs by suppressing Slug expression while increasing E-cadherin and β-catenin expression. In the same studies, HPV-negative head and neck SCCs were found to exhibit higher expression of KLF4 and Slug than the HPV-positive tumors, while HPV transcripts were shown to stabilize KLF4 at the protein level and upregulate E-cadherin. Taken together, those data suggest KLF4-mediated mechanism that suppresses EMT in HPV-positive head and neck SCCs [181,182]. Another study has elucidated the role of the cytoskeletal regulator, Mammalian Enabling Protein (Mena), in EMT, invasion, and metastasis spread in human oral SCCs [184]. It has been shown that Mena expression was considerably impaired in HPV16-positive oral SCCs compared to the HPV-negative ones; in the latter, Mena expression was associated with lymphatic metastases and EMT signatures (decreased E-cadherin, increased vimentin and MMP2) in contrast to HPV16-positive oral SCCs. Thus, integrated HPV16 is able to suppress Mena-dependent mechanisms of EMT in human oral SCCs [184]. Transmembrane protein 92 (TMEM92) expression was shown to be elevated in HPV-negative human head and neck SSCs compared to their HPV-positive counterparts, and high TMEM92 expression was associated with poor survival. Single-cell analysis revealed that TMEM92 promotes proliferation, migration, invasion, EMT, and cisplatin resistance in SCC cells [185].

In parallel with the existence of HPV infection-altered epigenetic regulators that promote EMT in oral cancer [94,173], certain shifts in microRNA spectra can exert inhibitory effects on EMT in HPV-positive head and neck SCCs. For example, the relatively higher levels of miR-34a in HPV-positive head and neck SCC cells may be responsible for the lower expression levels of EMT- and CSC-related transcription factors compared to the HPV-negative SCC cells [179]. Zhang et al., [180] reported the upregulation of miR-99a-3p and miR-4746-5p and downregulation of miR-411-5p in the tissue samples of HPV16-positive head and neck SCCs which was associated with the revealed suppression of the EMT/invasion gene set in those samples.

#### 3.2.3. Can EMT Markers Be a Prognostic Factor for Human Head and Neck Cancers?

If some of these HPV-associated mechanisms inhibiting EMT act in vivo, this may partly explain (at least for head and neck SCCs) the better treatment outcomes and favorable prognosis for patients with HPV-positive tumors. Indeed, if intratumoral EMT results in invasion, metastases, chemoresistance, and other complications that promote cancer progression, any (e.g., HPV-mediated) mechanisms that somehow suppress EMT will contribute to the better patient outcome. It is quite intriguing why, in contrast to the situation with HPV-positive cervical cancers where HPV infection clearly stimulates or promotes EMT (see Section 3.1), HPV integrated into head and neck SCCs can exert either EMT-promoting or EMT-suppressing effects. Perhaps such contrasting effects are due to the existence of different subtypes of head and neck SCC, which have different regulatory pathways in HPV-positive patients, so that their EMT programs are diversely modulated by HPV. Moreover, conflicting results may stem from differences in tumor location and size, HPV detection methods, p16 status, risk factors (smoking or alcohol consumption), treatment regimens, and patient characteristics. On the other hand, this discrepancy may have therapeutic applications: if there are alternative scenarios for the impact of HPV on EMT in head and neck SCCs, it seems possible to purposefully stimulate the HPV-related scenarios that suppress intratumoral EMT.

Attempts to use the detection of EMT in head and neck SCCs for prognoses yielded rather conflicting data. In some early publications, detectable EMT signatures in HPV-positive SCCs were shown to correlate with aggressive metastases, but at the same time with favorable prognosis [161,162]. Later, Kaneko et al. [168] found that EMT was associated with recurrence in HPV-positive head and neck SCCs. While some research groups associated EMT in HPV-positive head and neck SCCs with worst outcomes [166,186], other researchers found no relationship between the detection of EMT markers and survival among patients with HPV-positive oropharyngeal SCCs [165]. The revealed correlation between the high level of Snail1 expression in CTCs from patients with HPV-positive head and neck SCC and a superior prognosis for them [183] also explanation. Thus, the prognostic potential of EMT molecular signatures toward HPV-positive head and neck SCCs has not yet been determined.

#### 3.2.4. Approaches to Inhibiting EMT in Cells of Human Head and Neck Carcinomas

Despite the above contradictory data on the role of HPV in the mechanism of EMT in head and neck SCCs, the adverse consequences of EMT and its contribution to the tumor pathogenesis are not in doubt. Therefore, some research efforts are aimed at developing ways to inhibit EMT in head and neck cancers, including the HPV-positive cases. In particular, SAHA (Vorinostat), a clinically applicable inhibitor of histone deacetylase (HDAC), was shown to reverse EMT in both HPV-positive and HPV-negative head and neck cancer cell lines; the EMT-reversing effect was manifested in upregulation of E-cadherin and downregulation of vimentin in the SAHA-treated cell cultures, which was accompanied by an enhancement of the antiproliferative action of gefitinib on those human cancer cells [187]. Berggren et al. [188] reported that PF-3644022, an inhibitor of mitogen-activated protein kinase-activated protein kinase 2 (MK2), suppressed the radiation-stimulated EMT in both HPV-positive and HPV-negative human head and neck SCC cells. In that study, the MK2 inhibitor abrogated both the radiation-responsive phosphorylation of Hsp27 (a substrate of MK2 and regulator of F-actin dynamics) and the increase in EMT markers such as Snail1, Snail2, and vimentin in the in vitro irradiated SCC cell cultures, and also radiosensitized SCC xenografts growing in nude mice [188]. This is consistent with another finding that phosphorylated Hsp27 may stimulate EMT and increase migration in cells of human nasopharyngeal carcinoma [189]. The published data on inhibition of HDAC [187] and MK2 [188] suggest that EMT in both HPV-positive and HPV-negative head and neck cancers can be pharmacologically suppressed, and such inhibitory co-treatments may improve the therapeutic effects of gefitinib or radiation exposure. According to Lim et al. [170], partial EMT and invasion in HPV16-positive oropharyngeal SCCs may be suppressed using an SSO-based gene therapy tool that inhibits the formation of E6*I, the spliced isoform of HPV16 E6 responsible for partial EMT.

Overall, the interplay between HPV and EMT in head and neck cancers appears to be more complex than in cervical cancers. HPV-positive head and neck tumor cells exhibit a wide variety of mechanisms and pathways influencing EMT, and further rigorous research is needed to develop suitable methods for therapeutic targeting of intratumoral EMT or using its markers for prognosis.

### 3.3. Anal and Colorectal Cancers

Along with cervical cancer and oropharyngeal cancer, anal and colorectal cancers are thought to be the types of human malignancies that can be caused by HPV infection (see for a review [4,190,191]).

It has been suggested that high-risk HPV infection affects EMT-related genes and pathways, which promote the invasive and metastatic progression of carcinomas in the colorectal zone; there are data supporting this suggestion. In an in vitro study [192], the co-expression of E6/E7 of HPV16 and HPV18 in cells of the human colorectal carcinoma-derived cell lines HCT116 and HT29 caused the EMT-related changes in the expression of E-cadherin, fascin, and vimentin, as well as increased cell migration and invasion.

As for molecular analysis of the HPV-positive human tissue samples, Garcia et al. [85] identified multiple HPV-derived microRNAs in the biopsies of anal SCCs, as well as target genes of those microRNAs being implicated in intratumoral EMT and contributing to anal cancer progression. The authors suggested a potential utility of some of the revealed (HPV-associated) microRNAs for early diagnosis and/or therapeutic intervention of/in anal SCCs [85].

Of note, in the case of co-infection with HPV and EBV in colorectal cancer, oncoproteins of these two viruses can cooperate to trigger and/or enhance EMT in the doubly infected carcinoma cells, which may aggravate tumor invasion and metastases [193].

To summarize the data presented in this subsection, it can be noticed that the relationship between HPV and EMT in colorectal/anal cancers has not yet been sufficiently explored. Clearly, further in-depth studies are needed to identify the key drivers and mechanisms of EMT in HPV-related colorectal and anal carcinomas to use them for prognosis and/or molecularly targeted therapy.

### 3.4. Lung Cancers

Although definitive evidence of HPV-induced carcinogenesis in lungs is still lacking, causal links between HPV infection and the clinical picture of human lung adenocarcinomas and lung SCCs are gradually being established [8,194]. Moreover, there are data that HPV infection can promote EMT in human lung epithelium and lung carcinoma cells, which may be important for the development and pathogenesis of lung cancer (see Section 2.2.5 and refs [90,195,196,197]).

The fact that HPV induces EMT in lung cancer cells has been established in experimental models with human non-small cell lung cancer (NSCLC) cell lines. Zhang et al., [196] have found that the overexpression of HPV16 E6 and E7 in NSCLC cells promotes EMT-related morphology, downregulates the epithelial markers (E-cadherin, ZO-1), and upregulates the mesenchymal markers (N-cadherin, vimentin) and EMT-driving transcription factors (ZEB1, Snail1). The EMT induction was accompanied by STAT3 activation, while WP1066, a specific STAT3 inhibitor, reversed the effects of HPV16 E6 on the expression of ZO-1, vimentin, and ZEB1 in NSCLC cells. Those results suggest that HPV16 E6 and E7 oncoproteins can trigger or enhance EMT in NSCLC cells via the WP1066-sensitive activation of the STAT3 pathway [196]. Later, the same researchers found that HPV16 E7 enhances the expression of monoamine oxidase A (MAOA, a ROS-generating enzyme) in NSCLC cells, which leads to ROS generation and subsequent EMT with activated cell migration and invasion [197]. Notably, MAOA knockout inhibited HPV16 E7-driven migration, invasion, and EMT, and significantly reduced HPV16 E7-induced ROS generation in NSCLC cells in vitro, while inhibited tumor growth, metastases, and the expression of EMT markers (N-cadherin, Slug) induced by HPV16 E7 in NCI-H460 NSCLC subcutaneous xenograft and intrapulmonary models in nude mice [197]. These data delineate the crucial role of MAOA and ROS in HPV16 E7-induced EMT/metastases in NSCLC.

A later publication by the same research group showed that the exosomes (E7 Exo) secreted by HPV16 E7-overexpressing NSCLC cells enhance the migratory and invasive capabilities of NSCLC cells as compared with the exosomes from the empty vector-infected NSCLC cells [198]. E7 Exo induced EMT in both in vitro cultured NSCLC cells and subcutaneous NSCLC xenografts in nude mice, which was manifested in the respective changes in the expression of E/N-cadherin, vimentin, Snail1, Slug, and Twist1. Importantly, HPV16 E7 enhanced the expression of EGFR and its phosphorylated form in both NSCLC cells and the exosomes they secrete, while the inhibition of EGFR activation (with PD168393) or exosome secretion (with GW4869) suppressed E7 Exo-induced migration, invasion, and EMT in NSCLC cells [198]. Those findings indicate the involvement of EGFR-expressing exosomes in HPV16 E7-induced EMT of NSCLC cells, which may contribute to the pathogenesis of HPV-positive lung cancers.

The experimental data obtained with HPV16 E7-overexpressing NSCLC cells [196,197,198] are partly supported by in situ studies of human lung tissue samples [90,195]. Rezaei et al. [90] revealed the decreased expression of E-cadherin and the increased expression of N-cadherin, Slug, and Twist in HPV-positive tissue samples of human lung carcinomas compared to the HPV-negative samples, which implies a direct link between HPV positivity and EMT in lung cancer cells. The researchers also found an increase in inflammatory cytokines in the HPV-positive lung cancer samples and suggested that inflammation-induced EMT contributes to the development and progression of lung carcinomas [90]. In the next study [195], decreased E-cadherin along with increased N-cadherin and MMP2 were detected in the HPV-positive lung tumors compared to the HPV-negative lung tissue samples. The authors also described dramatic shifts in microRNAs expressed in the HPV-positive lung tissue samples and suggested that the HPV infection-induced alterations in microRNA spectra promote EMT that is involved in the development/progression of lung cancer [195]. In both studies [90,195], high-risk HPV16 was found to be the most prevalent genotype in the HPV-positive lung tissue samples explored.

Finalizing this subsection, it can be concluded that (i) HPV infection may be a risk factor for human lung carcinogenesis; (ii) HPV infection induces and/or promotes EMT in human lung epithelium and lung carcinoma cells, which may contribute to the development and pathogenesis of lung cancer; (iii) this HPV-associated EMT in lung cancer cells can be driven by different molecular mechanisms and factors including inflammatory cytokines, ROS, STAT3 activation, EGF/EGFR pathway, microRNA expression, and exosome secretion; (iv) the HPV-associated EMT in lung carcinomas can be prevented or impaired by inhibitors of STAT3, EGFR signaling, or exosome secretion, so that clinically applicable analogs of such inhibitors might be used for the treatment of HPV-positive lung cancers. Despite all this, more in-depth studies are clearly needed to evaluate a prognostic significance of EMT and effectively target it in HPV-positive lung cancers.

### 3.5. Other Cancers

There are a number of publications devoted to the interplay between HPV infection and EMT in tumors of various locations, different from the cancers described in Section 3.1, Section 3.2, Section 3.3 and Section 3.4.

At least some cases of vulvar cancer can be caused by high-risk HPVs [199]. Currently, there are only a couple of older publications linking vulvar cancer with both HPV and EMT [200,201], which argue that EMT is more likely to be associated with the HPV-negative tumors, whereas the HPV-positive tumors, having better prognosis and outcomes, progress through pathways independent of EMT. Those findings are in contrast to the published data on HPV-positive cervical cancers, where HPV infection has been shown to initiate and promote EMT through multiple mechanisms (see Section 3.1). It should be noted that those older (preliminary) data [200,201] have not been confirmed by any later publications, and it cannot be ruled out that new studies using more modern methods and approaches will yield different results.

Human malignancies that can be caused by HPV infection include penile cancer [202]. Equine penile SCCs are also associated with papillomavirus infection, and this model has been used to explore intratumoral EMT [203]. There it was found that E6 and E7 expression in papillomavirus-positive equine SCCs resulted in the induction of EMT with the downregulation of epithelial markers (E-cadherin, β-catenin and cytokeratin) and the upregulation of mesenchymal markers (N-cadherin and vimentin) as well as EMT-related transcription factors (Twist1, ZEB1) [203]. On the contrary, comparative analysis of the expression of E-cadherin, vimentin, and EMT-related transcription factors Twist, ZEB1, and Snail in the samples of HPV-positive and HPV-negative human SCCs has revealed that HPV positivity was associated only with the loss of membranous E-cadherin but not with the canonical EMT, which, in turn, was found to be associated with HPV-negative SCCs and poor prognosis [204]. Later, transcriptomic studies showed that HPV-positive human penile SCC cells exhibited a typical epithelial phenotype, while partial EMT was observed only in penile SCC cells with dysfunction of mutated p53 [205]. Such a discrepancy between equine and human penile SCCs is not yet fully understood, and further detailed studies are needed to clarify the contribution of HPV infection and EMT to the pathogenesis of human penile cancer.

Regarding non-melanoma skin cancers, in a rodent model with *Mastomys coucha*, cells of papillomavirus-associated cutaneous SCCs were found to undergo EMT with characteristic changes in the expression of cytokeratin, vimentin, and E-cadherin [206]. However, a potential role of EMT in the pathogenesis of HPV-positive skin cancers in humans has not yet been established.

Obviously, the data reviewed in this subsection are still preliminary and insufficient, and additional studies are needed to determine the role of EMT in the pathogenesis of HPV-positive cancers of the vulva, penis and skin in humans.

## 4. Conclusions and Perspectives

Despite the apparent success of HPV vaccination, millions of cases of HPV-related cancers are diagnosed worldwide every year. Since HPV-induced carcinogenesis remains a pressing issue, it is important to elucidate the underlying molecular mechanisms to develop new biomarkers and treatments for the diagnosis and prevention of HPV-caused cancers.

From the data in Section 2 it follows that, firstly, HPV oncoproteins E6, E7 and E5 stimulate EMT in the HPV-infected human epithelium and, secondly, this HPV infection-induced EMT appears to contribute to malignant transformation of the HPV-infected cells to occur. In this case, promising directions appear to be: (i) the development of convenient methods for determining EMT markers in patients’ lesions caused by HPV infection, and (ii) the search for and use of suitable inhibitors to target EMT in those lesions to prevent the EMT-dependent, HPV-associated tumorigenesis (see Figure 3). Regarding the first point, PCR-based tests (similar to those used to detect HPV in patients’ scraping samples) could be used to quantify EMT markers at the mRNA level. PCR detection of EMT likely requires more cellular material than is obtained via scrapings, as well as additional cytological analysis to distinguish epithelial cells that have undergone EMT from genuine mesenchymal cells, which are expected to be present in HPV-induced lesions. As for the second point, to select a suitable, clinically applicable inhibitor of EMT, it is necessary to perform a preliminary (focused) screening of various potentially promising compounds and drugs; for this purpose, appropriate in vitro test systems with HPV-positive human normal epithelial cell lines should be created and exploited to assess the reversal of EMT in them after inhibitory treatments. Overall, the idea of inhibiting EMT in patients’ LSILs and HSILs to lower risk of cancer development seems sound and potentially feasible but additional studies and clinical trials are certainly needed to validate it.

Summarizing the data reviewed in Section 3, one can note the enormous variety of molecular factors, pathways, and axes influencing EMT in HPV-positive cancers. The diversity of HPV-mediated EMT mechanisms in human malignancies of different locations is also clearly evident in those data. Although HPV detection in patients’ tumors is usually associated with a favorable prognosis/outcome, intratumoral EMT worsens the pathogenesis of both HPV-positive and HPV-negative carcinomas, as EMT results in cancer cell invasion, metastasis spread, and resistance to many agents of conventional anticancer therapy (see also Figure 2). Thus, the task of inhibiting intratumoral EMT seems extremely relevant regardless of HPV status of the target tumor. In parallel with inhibiting or reversing intratumoral EMT, its consequences such as cancer cell migration and cancer cell drug resistance must also be addressed.

According to the data reviewed in Section 3.1, there are many various HPV-dependent mechanisms that initiate and/or promote EMT in human cervical cancers. Therefore, there is potential for multiple interventions targeting EMT in HPV-positive cervical carcinomas to prevent or minimize the above disease-aggravating consequences of the HPV-associated intratumoral EMT. The data in Table 1 show that there are a number of small molecule compounds that can inhibit or reverse EMT in HPV-positive cervical cancer cells, several of which are approved antitumor chemotherapeutic agents, and one is even recommended for the treatment of cervical cancer. In this regard, the next objective is to conduct more advanced preclinical studies aimed at inhibiting EMT in in vivo models with HPV-positive cervical cancer xenografts, in order to move closer to clinical trials.

Unlike the situation with human cervical cancer, HPV positivity in human head and neck carcinomas does not always indicate stimulation of intratumoral EMT via HPV-dependent mechanisms. The reviewed papers on HPV and EMT in head and neck cancer demonstrate a duality: there are HPV-related mechanisms that promote EMT, and there are HPV-related mechanisms that suppress EMT (see Section 3.2). The latter mechanisms appear to contribute to the better outcomes that are attributed to most HPV-positive oropharyngeal malignancies. There appears to be a need for test systems and protocols for detecting EMT markers in biopsy obtained from patients with HPV-positive head and neck carcinomas to select optimal treatment strategies. When analyzing EMT markers, it should be taken into account that increased vimentin expression in tumors may indicate not EMT, but the intratumoral accumulation of mesenchymal cells such as CAFs, vascular cells, and immune cells. In turn, the presence of E-cadherin in the tested sample may not mean the absence of EMT, but rather the implementation of partial (hybrid) EMT, which is associated with even more active metastasis spread and an even worse outcome. Ideally, the test system should be able to distinguish between complete EMT and partial EMT in the harvested biopsy specimens. Identification and subsequent targeting of intratumoral EMT may be beneficial for the treatment of HPV-negative head and neck cancers.

Meanwhile, several publications indicate that the presence of HPV in human lung cancer cells is associated with the implementation of EMT in them (see Section 3.4), which is reminiscent of the situation with cervical cancer, although it has not yet been proven that human lung cancer can be caused by HPV infection. Clearly, these studies should be continued to precisely determine the relationship between HPV and EMT in the development/pathogenesis of human lung cancers.

Currently, there are too few publications to draw any definitive conclusions regarding the role of EMT in the pathogenesis of HPV-positive skin, vulvar, and penile cancers. Further research on human tumors with such locations is clearly needed herein.

The limitations of the current evidence include the predominance of in vitro studies, heterogeneity in the definition and measurement of EMT, the presence of genuine mesenchymal cells in tissues alongside epithelial cells that have acquired a mesenchymal phenotype via EMT, reliance on individual EMT markers in some studies, differences between HPV genotypes and tumor locations, limited prospective clinical validation, and the difficulty of establishing causality based on human observational data.

In conclusion, two key objectives, namely, timely detection and inhibition of EMT, appear relevant for addressing both the problems of (i) prevention and (ii) treatment of HPV-associated cancers. The authors hope that this review will encourage researchers to focus their efforts on these objectives.

## Figures and Tables

**Table 1:** Agents that have been shown to inhibit or revert EMT in HPV-positive human cervical cancer cells and also exert beneficial (antitumor) effects.

Cells or Tumor	HPV Status	EMT-Inhibiting Agent	Achieved Effects	Refs
SiHa	HPV16+	Curcumin	Increased E-cadherin; decreased N-cadherin, vimentin and Slug; suppressed migration	[122]
HeLa	HPV18+	RKI-1447 ^1^ (MSX1-OE ^2^)	Inhibition of MSX1-induced EMT; reversal of migratory phenotype; reduction in filopodia	[128]
SiHa	HPV16+	A1E ^3^	Increased E-cadherin; decreased vimentin, β-catenin and OCT3/4; suppressed cell migration; reduced expression of cancer-stemness markers	[144]
HeLa SiHa	HPV18+ HPV16+	Adenosine	Increased E-cadherin; Decreased N-cadherin, Snail and fibronectin; suppressed migration and invasion; stimulated apoptosis	[145]
HeLa CaSki C33A	HPV18+ HPV16+ HPV−	Hookworm *N. brasiliensis* L3-antigen	Reduced expression of vimentin and N-cadherin; decreased migration in both HPV+ and HPV− cells	[146]
HeLa CaSki SiHa (in vitro and xenograft in nude mice)	HPV18+ HPV16+ HPV16+	Bazedoxifene	Increased E-cadherin; decreased vimentin, β-catenin and Wnt5β; inhibited EMT signaling; Suppressed migration; stimulated apoptosis; repressed tumor growth in vivo	[147]
HeLa	HPV18+	*Caulerpa racemosa*	Increased E-cadherin; decreased Snail and vimentin; suppressed cell migration	[148]
ME180 C33A	HPV68+ HPV−	EGCG ^4^	Prevention of EMT; reduced expression of MMP2; suppressed cell migration and clonogenicity in both HPV+ and HPV− cell lines	[149]
HeLa	HPV18+	Oxyresveratrol	Decreased vimentin, N-cadherin and β-catenin; retarded migration; enhanced apoptosis	[150]
HeLa SiHa ME180	HPV18+ HPV16+ HPV68+	Plumbagin	Increased E-cadherin; decreased N-cadherin, vimentin, β-catenin and MMP2/9; suppressed migration and invasion; enhanced apoptosis	[151,152]
SiHa	HPV16+	HucMSC-exosomes ^5^	Increased E-cadherin; decreased N-cadherin and Snail; suppressed migration; attenuation of modeled inflammation	[153]
HeLa (in vitro and xenograft in nude mice)	HPV18+	Bevacizumab	Increased E-cadherin; decreased N-cadherin, Snail and Notch1; suppressed migration; sensitization to cisplatin	[154]
SiHa CaSki HeLa C33A	HPV16+ HPV16+ HPV18+ HPV−	ZHPV16E7-GrB ^6^	Reversal of EMT in HPV16+ cells only; increased E-cadherin; decreased N-cadherin, vimentin and Snail; caspase-3/8 activation; stimulated apoptosis and pyroptosis	[155]
C33A SiHa HeLa	HPV− HPV16+ HPV18+	ZHPV16-18E7-GrB ^7^	Reversal of EMT in HPV16+ and HPV18+ cells; increased E-cadherin; decreased vimentin and Snail; suppressed cell migration; activation of caspase-3/8 and apoptosis; repressed tumor growth in vivo	[156]

^1^ RKI-1447—a selective inhibitor of ROCK (Rho-associated protein kinase) enzymes; ^2^ HeLa cells overexpressing MSX1; ^3^ A1E—a mixture composed of 11 oriental medicinal herbs; ^4^ EGCG—epigallocatechin gallate; ^5^ HucMSC-exosomes—human umbilical cord mesenchymal stem cells-derived exosomes; ^6^ ZHPV16E7-GrB—a fusion construct of an HPV16E7-specific affibody (ZHPV16E7) with the cytotoxic immune effector granzyme B (GrB); ^7^ ZHPV16-18E7—a fusion construct of a bispecific affibody simultaneously targeting the early oncogenic proteins E7 of HPV16 and HPV18 types with the cytotoxic immune effector GrB.

## Data Availability

No new data were created in this study. Data sharing is not applicable to this review article.

## Abbreviations

The following abbreviations are used in this manuscript:

ABCG2	ATP-binding cassette (ABC) membrane transporter G2
ALDH	Aldehyde dehydrogenase
CAF	Cancer-associated fibroblast
CIN	Cervical intraepithelial neoplasia
CSC	Cancer stem cell
CTC	Circulating tumor cell
DAMP	Damage-associated molecular patterns
EBV	Epstein–Barr virus
ECM	Extracellular matrix
EGCG	Epigallocatechin gallate
EGF	Epidermal growth factor
EGFR	Epidermal growth factor receptor
EMT	Epithelial-to-mesenchymal transition
FGF	Fibroblast growth factor
FGFR	Fibroblast growth factor receptor
HIF	Hypoxia-inducible factor
HIV	Human immunodeficiency virus
HPV	Human papillomavirus
HSIL	High-grade squamous intraepithelial lesion
Hsp	Heat shock protein
IL	Interleukin
LSIL	Low-grade squamous intraepithelial lesion
LPS	Lipopolysaccharide
MAPK	Mitogen-activated protein kinase
MET	Mesenchymal-to-epithelial transition
MMP	Matrix metalloproteinase
NSCLC	Non-small cell lung cancer
PD-L1	Programmed death receptor ligand 1
PI3K	Phosphoinositide 3-kinase
ROS	Reactive oxygen species
SCC	Squamous cell carcinoma
SMA	Smooth muscle actin
TGFβ	Transforming growth factor β
TGFβ R	Transforming growth factor β receptor
TME	Tumor microenvironment
TNF	Tumor necrosis factor
VEGF	Vascular endothelial growth factor
